# Chronic cigarette smoking is linked with structural alterations in brain regions showing acute nicotinic drug-induced functional modulations

**DOI:** 10.1186/s12993-016-0100-5

**Published:** 2016-06-02

**Authors:** Matthew T. Sutherland, Michael C. Riedel, Jessica S. Flannery, Julio A. Yanes, Peter T. Fox, Elliot A. Stein, Angela R. Laird

**Affiliations:** Department of Psychology, Florida International University, AHC-4, RM 312, 11200 S.W. 8th St, Miami, FL 33199 USA; Department of Physics, Florida International University, Miami, FL USA; Department of Psychology, Auburn University, Auburn, AL USA; Research Imaging Institute, University of Texas Health Science Center, San Antonio, TX USA; South Texas Veterans Health Care System, San Antonio, TX USA; State Key Laboratory for Brain and Cognitive Sciences, University of Hong Kong, Hong Kong, China; Neuroimaging Research Branch, National Institute on Drug Abuse, Intramural Research Program, NIH/DHHS, Baltimore, MD USA

**Keywords:** Cigarettes, Nicotine, Addiction, Gray matter, Morphometry, Insula, Mediodorsal thalamus, Ventromedial prefrontal cortex, Cerebellum

## Abstract

**Background:**

Whereas acute nicotine administration alters brain function which may, in turn, contribute to enhanced attention and performance, chronic cigarette smoking is linked with regional brain atrophy and poorer cognition. However, results from structural magnetic resonance imaging (MRI) studies comparing smokers versus nonsmokers have been inconsistent and measures of gray matter possess limited ability to inform functional relations or behavioral implications. The purpose of this study was to address these interpretational challenges through meta-analytic techniques in the service of clarifying the impact of chronic smoking on gray matter integrity and more fully contextualizing such structural alterations.

**Methods:**

We first conducted a coordinate-based meta-analysis of structural MRI studies to identify consistent structural alterations associated with chronic smoking. Subsequently, we conducted two additional meta-analytic assessments to enhance insight into potential functional and behavioral relations. Specifically, we performed a multimodal meta-analytic assessment to test the structural–functional hypothesis that smoking-related structural alterations overlapped those same regions showing acute nicotinic drug-induced functional modulations. Finally, we employed database driven tools to identify pairs of structurally impacted regions that were also functionally related via meta-analytic connectivity modeling, and then delineated behavioral phenomena associated with such functional interactions via behavioral decoding.

**Results:**

Across studies, smoking was associated with convergent structural decreases in the left insula, right cerebellum, parahippocampus, multiple prefrontal cortex (PFC) regions, and the thalamus. Indicating a structural–functional relation, we observed that smoking-related gray matter decreases overlapped with the acute functional effects of nicotinic agonist administration in the left insula, ventromedial PFC, and mediodorsal thalamus. Suggesting structural-behavioral implications, we observed that the left insula’s task-based, functional interactions with multiple other structurally impacted regions were linked with pain perception, the right cerebellum’s interactions with other regions were associated with overt body movements, interactions between the parahippocampus and thalamus were linked with memory processes, and interactions between medial PFC regions were associated with face processing.

**Conclusions:**

Collectively, these findings emphasize brain regions (e.g., ventromedial PFC, insula, thalamus) critically linked with cigarette smoking, suggest neuroimaging paradigms warranting additional consideration among smokers (e.g., pain processing), and highlight regions in need of further elucidation in addiction (e.g., cerebellum).

**Electronic supplementary material:**

The online version of this article (doi:10.1186/s12993-016-0100-5) contains supplementary material, which is available to authorized users.

## Background

Over the past two decades, neuroimaging has contributed important insight into the structural and functional brain alterations linked with drug abuse in general [1–3] and nicotine addiction in particular [4–6]. For example, such studies have revealed that nicotine administration alters functional brain activity, inducing enhanced activity in some regions involved with attention and cognition (e.g., thalamus, lateral frontoparietal cortices, anterior cingulate cortex [ACC]) yet reducing activity in other regions involved with task-irrelevant mental operations (e.g., mind wandering; ventromedial prefrontal cortex, posterior cingulate cortex, parahippocampus) [6–8]. These functional brain alterations may, in part, provide a neurobiological account of the well-documented cognitive enhancing properties of acute nicotine administration [9, 10]. On the other hand, chronic cigarette smokers, compared with nonsmokers, exhibit poorer global cognition and impaired performance on specific measures of working memory, cognitive flexibility, visuospatial learning and memory, and processing speed [11–13]. Aligning with such neurocognitive observations, structural magnetic resonance imaging (MRI) studies have detected reduced gray matter integrity among smokers in multiple discrete brain regions including the prefrontal cortex (PFC), ACC, insula, thalamus, and cerebellum [e.g., 14–17]. Such regional atrophy may result from the deleterious impact of cigarette smoking and/or reflect predisposing neurobiological, neurocognitive, or personality factors.

However, structural MRI results among chronic smokers, to some degree, have been inconsistent. For example, gray matter in the insula of smokers has been reported to be decreased [15, 18, 19], increased [20], or comparable to that among nonsmokers [17, 21]. Similarly, whereas some studies have detected smoking-related structural decreases in the ventromedial PFC [17, 19, 22] or thalamus [17, 21, 23], others have not [14, 21, 24]. Heterogeneous findings may be the product of cross-sectional designs with modest-to-moderate sample sizes, between-study variability in participant attributes (e.g., varying age ranges, smoking histories, sex ratios, or other sociodemographic characteristics), and/or methodological differences in MRI acquisition or data analysis parameters (e.g., smoothing, registration techniques, or normalization templates). Such issues constrain interpretations from single studies and necessitate the post hoc integration of results from multiple independently conducted studies to better estimate parameters of interest [25]. Accordingly, neuroimaging meta-analytic techniques have been increasingly adopted to delineate spatially convergent results across studies, including identification of consistently observed gray matter alterations among specific phenotypes [26–28].

Although useful for elucidating potential structural alterations among smokers, measures of gray matter are limited in their ability to inform interpretation of functional relations or behavioral implications. Regarding structural–functional relations, a plausible hypothesis is that brain regions showing chronic smoking-related structural alterations overlap those same regions showing acute nicotinic drug-induced functional modulations. One perspective is that the repeated impact of nicotine exposure within discrete brain regions over an individual’s extended smoking history culminates in neuroadaptations that may manifest as gray matter perturbations in those same regions. An alternative perspective is that pre-existing structural alterations may render some individuals more susceptible to acute pharmacologic effects and, in turn, to addiction. Regardless of the causative pathway (or combination thereof), integrating structural and functional neuroimaging results may provide insight into the neurobiological processes potentially contributing to the initiation, escalation, and/or maintenance of cigarette smoking. In other words, a multimodal perspective may allow for enhanced interpretation of structural alterations [29, 30]. Regarding structural-behavioral implications, a frequently posed question not easily answered by considering morphometric outcomes is: what are the behavioral consequences of structural alterations in a particular brain region (or set of regions). As opposed to conjectural discussion of behavioral relevance, empirical approaches to more fully contextualize gray matter alterations are of growing interest [e.g., 31–33]. Such approaches provide an objective means to support behavioral interpretations and/or suggest neuroimaging paradigms warranting additional consideration among a particular phenotype.

In the current study, we aimed to address these interpretational challenges by employing established and emergent meta-analytic techniques to clarify the impact of cigarette smoking on gray matter integrity and to more fully contextualize such morphometic alterations. The specific goals of our study were threefold. First, we sought to identify convergent structural alterations across studies associated with chronic smoking via the well-established, coordinate-based activation likelihood estimation (ALE) meta-analytic framework. We operationalized the effects of chronic smoking as gray matter alterations identified in studies utilizing smoker versus nonsmoker comparisons. Second, we tested the structural–functional hypothesis that chronic smoking-related gray matter alterations overlap those same regions showing acute nicotinic drug-induced functional effects via a multimodal meta-analytic assessment. Whereas we operationalized *chronic effects* as structural alterations identified in smoker versus nonsmoker (i.e., between-subjects) comparisons, we operationalized the *acute effects* of nicotinic acetylcholine receptor (nAChR) agonist administration as functional alterations indentified in pharmacological neuroimaging studies, the vast majority of which employed within-subjects (i.e., drug versus control condition) comparisons. Third, we sought to provide enhanced structural-behavioral insight via emergent database driven meta-analytic tools, which allow for the characterization of typical patterns of task-based co-activation and associated behavioral phenomenon for user-specified seed regions of interest. Specifically, using smoking-related gray matter alterations to define seed regions, we performed meta-analytic connectivity modeling [33] and behavioral decoding assessments [34, 35] on data archived in an extensive neuroimaging repository (http://www.brainmap.org/) to objectively support behavioral interpretations of structural alterations.

## Methods

### Structural MRI study search and selection

We performed an iterative literature search to compile structural neuroimaging studies interrogating gray matter alterations among chronic cigarette smokers compared with nonsmokers. In the first iteration, we searched the *Web of Science* (http://www.webofknowledge.com) and *PubMed* (http://www.pubmed.gov) databases for peer-reviewed articles with the following logical conjunction of terms: (“voxel-based morphometry” OR “morphometry” OR “gray matter density” OR “gray matter volume”) AND (“nicotine” OR “tobacco” OR “cigarette” OR “smok*”). In a second iteration, we consulted the bibliographies of recent review articles [5, 36] and one existing meta-analysis [37] for studies potentially not identified by the database queries. Although a previous meta-analysis has considered the structural impact of chronic smoking, we note that several additional studies have emerged subsequent to that report and highlight our emphasis on structural–functional and structural-behavioral relations as a further distinguishing characteristic. In a final iteration, we tracked the references of and citations to relevant papers, thereby compiling additional studies.

We included studies in this meta-analysis that: (1) assessed gray matter using structural MRI, (2) reported a set of coordinates (i.e., foci) from a between-subjects contrast comparing smokers to matched nonsmoking participants, (3) reported coordinates in a defined stereotaxic space (i.e., Talairach or Montreal Neurological Institute [MNI]), (4) performed a whole-brain analysis, and (5) provided sufficient information regarding characterization of smoking behaviors (e.g., pack-years, Fagerström Test of Nicotine Dependence [FTND] scores, years smoking, number of cigarettes smoked per day), basic demographics of the study samples (e.g., age, sex, *N*), and data analysis strategies (e.g., smoothing parameters, statistical thresholds).

Accordingly, we identified 15 peer-reviewed articles involving 761 cigarette smokers and 1182 nonsmokers (Additional file 1: Figure S1; Table S1) [14, 15, 17–24, 38–42]. Across these 15 identified studies, the smoker samples were on average 41.8 ± 16.2 (mean ± SD) years of age and were composed of 40.9 ± 25.6 % females. At the time of scanning, smokers reported cigarette use for 22.9 ± 17.0 years, smoked 17 ± 4.0 cigarettes per day, and were moderately nicotine dependent as indicated by FTND scores (4.5 ± 1.3 out of 10). These characteristics were rather consistent across studies and are generally representative of community-based samples of smokers. The nonsmoker samples did not differ from smokers in terms of age (41.1 ± 17.5 years; *t*[14] = −0.9, *p* = 0.4) or sex (41.6 ± 25.0 % female: *t*[13]^1^ = 0.5, *p* = 0.6). Most studies mitigated the influence of other drug use by screening via interview and/or urine toxicology on scan days (11 of 15 studies; Additional file 1: Table S2). For each study, we also tabulated information on the type of MRI scanner used and data collection/analysis parameters (Additional file 1: Table S2). All included studies utilized significance thresholds corrected for multiple comparisons or uncorrected thresholds combined with a spatial extent criterion. These studies distinguished gray matter alterations by the nonsmoker > smoker (i.e., smoking-related decreases) and smoker > nonsmoker directions (i.e., smoking-related increases). Of those included, 14 studies (78 foci) reported gray matter decreases among smokers and 5 studies (10 foci) reported increases. Given the limited number of studies and recent arguments that ALE meta-analyses based on less than 10 experiments/studies run the risk of obtaining results driven by a single experiment as opposed to identifying convergence across experiments [43], gray matter increases were not considered further.

### Structural impact of chronic cigarette smoking: meta-analytic procedures

To identify areas of convergent gray matter decreases across studies, we performed a coordinate-based meta-analysis using the revised version [44, 45] of the activation (in this application, Anatomic) likelihood estimation (ALE) algorithm [26, 46] as implemented in *GingerALE v2.3.4* (http://www.brainmap.org/ale/). ALE is a voxel-wise approach for combining neuroimaging results across a collection of experiments/contrasts and thereby identifying locations of statistically significant spatial convergence. The ALE framework models foci as centers of three-dimensional Gaussian probability distributions, thus accounting for spatial uncertainty due to within- and between-study variability. Foci are weighted by study sample size, where larger samples are associated with narrower distributions and smaller samples with wider distributions. We first linearly transformed foci reported in MNI to Talairach space [47] and then generated modeled maps of each individual contrast using their respective foci (paralleling the modeled activation maps of functional MRI [fMRI] meta-analyses). Next, we calculated a voxel-wise ALE score (i.e., the union of all contrasts’ modeled maps) quantifying the spatial convergence of structural alterations across the brain. To identify clusters of statistically significant convergence, we compared these obtained ALE scores with those from an empirical null-distribution derived from a permutation procedure [27]. This comparison resulted in nonparametric *p* value maps, which we then thresholded at a cluster-corrected level (*p*_*corrected*_ < 0.05; voxel-level: *p* < 0.005, cluster extent: 344 mm^3^) and exported to *MANGO* (http://www.ric.uthscsa.edu/mango/) for visualization on an anatomical (Talairach) template.

### Conjoint chronic smoking-related structural effects and acute drug-induced functional effects: multimodal meta-analytic procedures

We leveraged previous meta-analytic outcomes regarding the impact of acute nAChR agonist exposure on brain function to enhance interpretation of structural alterations observed among chronic cigarette smokers. Specifically, in a previous meta-analysis [6] we identified 38 pharmacological fMRI studies that assessed the acute functional effects of nAChR agonist administration (i.e. pharmacologic administration or cigarette smoking) relative to a baseline condition (i.e., placebo administration or smoking abstinence condition) across various cognitive and affective neuroimaging paradigms. The studies meeting selection criteria in that functional meta-analysis involved 796 participants, reported 364 foci from 77 contrasts, and distinguished functional activity modulations by the baseline > drug (i.e., activity decrease) and drug > baseline (i.e., activity increase) directions. We characterized the impact of nAChR agonists on brain function using the ALE framework (paralleling that described above) and separately identified brain regions showing either convergent activity increases or decreases using a cluster-level corrected threshold (*p*_*corrected*_ < 0.05).

To test the hypothesis that smoking-related gray matter decreases overlap those same regions showing acute drug-induced effects, we conducted a multimodal meta-analytic assessment. Specifically, we performed a conjunction analysis to identify those brain regions, if any, showing statistically significant convergence when considering both: (1) chronic smoking-related structural effects (smokers versus nonsmokers), and (2) acute drug-induced functional effects (nAChR agonist manipulation versus control condition). Employing a conservative minimum statistic conjunction [48], we identified brain regions showing conjoint structural and functional effects by computing the intersection of the two thresholded meta-analytic maps combined with an additional overlap-cluster extent criterion (100 mm^3^).

### Behavioral relevance of structurally impacted regions: meta-analytic connectivity modeling and behavioral decoding

As gray matter assessments possess limited ability to inform functional or behavioral interpretations, we subsequently employed emergent meta-analytic tools to more fully contextualize the brain circuit-level and behavioral consequences of structural alterations identified among smokers [28, 49, 50]. Specifically, to determine whether structurally impacted brain regions reflect disruption of functionally interrelated neurocircuits, we utilized *meta-analytic connectivity modeling* (MACM), a validated database driven approach for delineating brain areas that co-activate with a seed region of interest (ROI) across many neuroimaging tasks [33, 51, 52]. This assessment was conducted using the BrainMap database (http://www.brainmap.org/) which is an online repository of over 13,500 neuroimaging contrasts from ~2800 journal articles (as of January, 2016) archived as three-dimensional coordinate-based results (*x*, *y*, *z*) as well as relevant metadata describing the associated experimental design [53–55]. Whereas we utilized the database’s archived activation coordinates to characterize the co-activation/connectional profile of structurally-identified ROIs, we utilized the metadata to facilitate behavioral interpretation of smoking-related structural alterations via *meta*-*analytic behavioral decoding*. Although the data utilized in these assessments were from healthy participants, they nonetheless offer a useful path to enhance interpretation of observed structural alterations among smokers. These analyses attempt to identify within a typical range of function whether pairs of regions interact and, if so, under what behavioral context. If two brain regions are structurally impacted by a certain neuropsychiatric condition and those same regions also appear to interact among healthy participants under a specific behavioral context, one plausible inductive conclusion is that the psychological processes associated with that behavioral context may be disrupted in the neuropsychiatric condition.

Moving beyond isolated regions, we conducted a MACM assessment to characterize the typical pattern of task-based, whole-brain co-activation for each of the structurally-identified ROIs. A MACM assessment aims to identify, across a domain-arching pool of studies interrogating various mental operations and task paradigms, brain areas that simultaneously co-activate with a user-specified seed region. In other words, a MACM assessment identifies brain areas most likely to be activated across all tasks, given activation within a seed. Similar to seed-based resting-state functional connectivity assessments of fMRI data, a MACM identifies those regions that are significantly related to, and presumably interact with the seed. First, we identified experiments in the database that reported one or more activation coordinates within a seed ROI using the *Sleuth* software application (http://www.brainmap.org/sleuth/). The seeds were 8 mm radius spheres centered on the voxels with maximum ALE values within each of the smoking-related gray matter loss regions identified above. We conducted separate searches and computed separate MACMs for each of these ROIs. As practiced in previous MACM assessments to achieve sufficient power [e.g., 56], only those ROIs associated with 30 or more experiments in the database were considered for further analyses. We employed 8 mm radial spheres to equate each ROIs volume and to seek a balance between returning a sufficient number of experiments from the database and minimizing overlap between ROIs that were in close proximity. Next, we extracted the whole-brain coordinates of all foci that co-activated with the seed, constraining this extraction to only activation foci (i.e., no deactivations) reported in studies examining healthy participants (i.e., no intervention or group comparisons). After converting foci reported in MNI to Talairach space [47], we supplied these foci as input to the ALE methodology described above thereby delineating regions of convergent co-activation with the seed when employing a cluster-corrected threshold of *p*_*corrected*_ < 0.05 (voxel-level: *p* < 0.001). These thresholded MACM maps for each ROI represent the above-chance probability that identified voxels co-activated with the respective seed across many neuroimaging tasks. To determine whether smoking-related gray matter loss regions reflected disruption of functionally interrelated neurocircuits, we quantified the degree to which one ROI’s MACM map intersected with any of the other structurally impacted ROIs. If one ROI’s MACM map overlapped at least 50 voxels of any other ROI, those two regions were considered to constitute a circuit-level functional interaction.

In addition to the co-activation coordinates, we also extracted the corresponding BrainMap metadata allowing for the generation of behavioral profiles for each pair of co-activating, and presumably functionally-related, ROIs. The metadata in the database are coded according to a well-defined taxonomy (http://www.brainmap.org/taxonomy/) cataloguing each contributing study’s experimental design, stimulus type, behavioral domain (and subcategory), and paradigm class [57, 58]. Under this taxonomy, behavioral domains (BD) represent the mental processes interrogated by the primary study’s statistical contrasts and comprise the main categories of *action*, *cognition*, *emotion*, *interoception*, and *perception* as well as BD subcategories (BD-S; e.g., *perception: somesthesis*-*pain*). Paradigm classes (PC) further categorize the specific task employed (e.g., *pain monitoring/discrimination*, *Go/No*-*Go*). We used these metadata terms to delineate behavioral phenomena linked with the concurrent activation of those pairs of structurally-identified ROIs considered to constitute circuit-level functional interactions in the above MACM assessments.

Specifically, we created behavioral profiles for circuits of interest by performing forward and reverse inference analyses [34, 35] on the associated distribution of metadata terms [59, 60]. In the forward inference approach, we tested whether the conditional probability of brain activation given a particular behavioral phenomenon (i.e., BD, BD-S, or PC), *p*(Activation|Phenomenon), was higher than the baseline probability of brain activation, *p*(Activation). Baseline activation was defined as the probability of finding a random activation from the database in the region(s) of interest. Significance was established with a binomial test (*p*_*FDR*-*corrected*_ < 0.05). In the reverse inference approach, we identified the most likely behavioral phenomenon (i.e., BD, BD-S, or PC) given activation in a region. This likelihood, *p*(Phenomenon|Activation), was derived from *p*(Activation|Phenomenon) as well as *p*(Phenomenon) and *p*(Activation) using Bayes’ Rule. Significance was established with a Chi-squared test (*p*_*FDR*-*corrected*_ < 0.05). Only BD, BD-S, and PC terms that were significant in both the forward and reverse inference approaches are reported.^2^

## Results

### Structural impact of chronic cigarette smoking

**Fig. 1:**
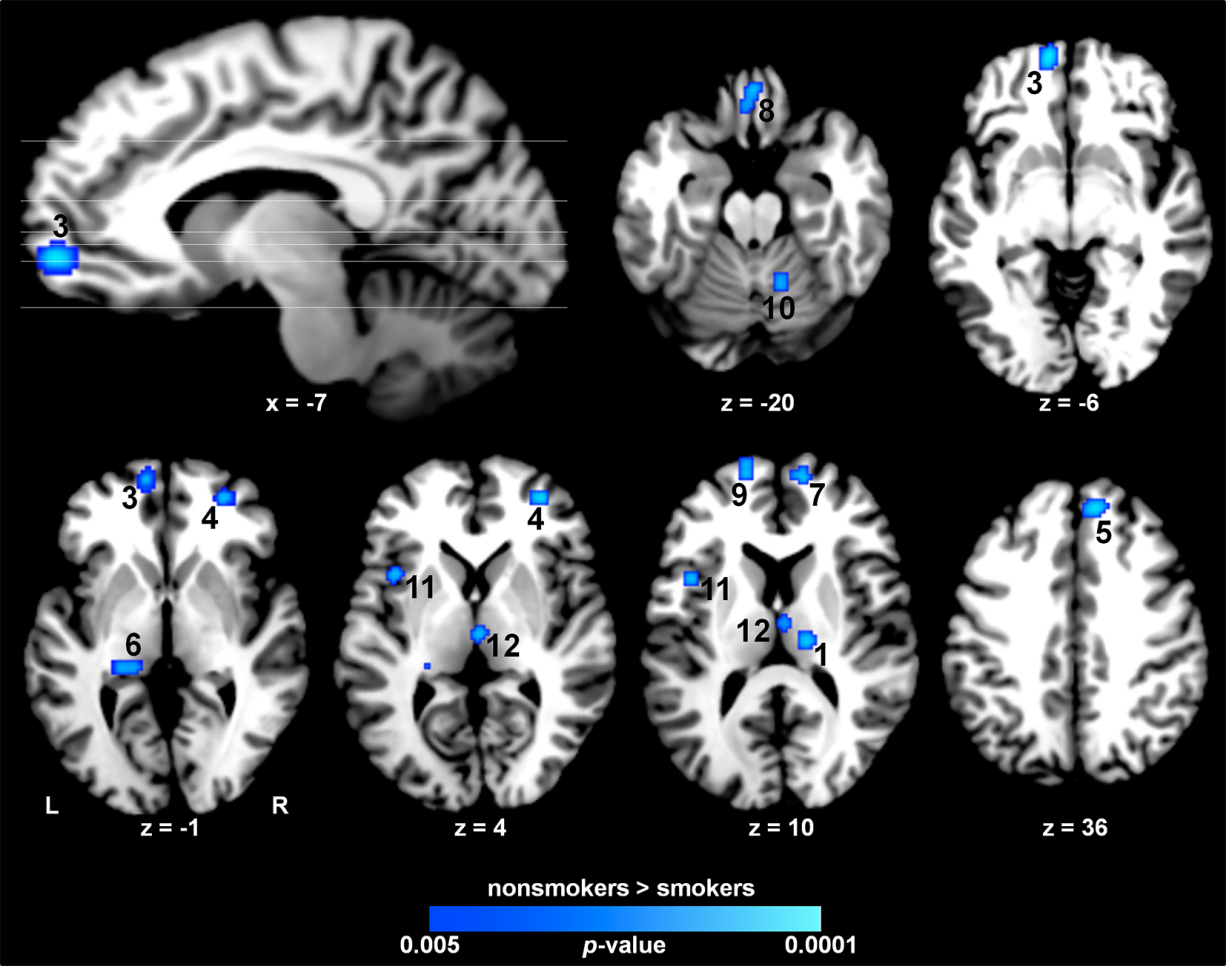
Structural impact of chronic cigarette smoking. Convergent gray matter decreases among smokers (nonsmokers > smokers) were observed notably in multiple PFC regions, the left insula, thalamus, and cerebellum. *Numbering* corresponds to coordinates listed in Table 1

To elucidate structural alterations associated with an extended smoking history, we conducted a meta-analysis identifying consistent gray matter decreases among smokers. This meta-analysis included 78 distinct foci from 14 peer-reviewed studies involving a total of 750 smokers and 1073 nonsmokers (Additional file 1: Table S1). Across these studies, ALE revealed convergent gray matter decreases in 12 distinct clusters, notably in the ventromedial PFC (vmPFC), left insula, and mediodorsal (MD) thalamus, as well as in the medial orbitofrontal cortex (mOFC), ventrolateral PFC (vlPFC), dorsomedial PFC (dmPFC), medial PFC (mPFC), left parahippocampal gyrus, and right cerebellum (Fig. 1; Table 1).

**Table 1:** Convergent gray matter decreases associated with chronic smoking: cluster coordinates

Cluster	Region		Volume	X	Y	Z
Nonsmokers > smokers						
1	Thalamus (lateral posterior nucleus)	R	592	16	−22	14
2	dmPFC (BA 6) (superior frontal gyrus)	R	576	16	24	52
3	vmPFC (BA10) (superior frontal gyrus)	L	568	−8	56	−4
4	vlPFC (BA 10) (middle frontal gyrus)	R	480	32	46	4
5	dmPFC (BA 8) (medial frontal gyrus)	R	480	10	40	36
6	Parahippocampal gyrus	L	432	−20	−34	0
7	mPFC (BA10) (medial frontal gyrus)	R	416	12	58	8
8	Medial OFC (BA 11)	B	384	2	34	−20
9	mPFC (BA10) (medial frontal gyrus)	L	376	−14	58	8
10	Cerebellum (dentate)	R	368	14	−58	−20
11	Insula (BA 13)	L	368	−40	8	10
12	Thalamus (medial dorsal nucleus)	B	360	2	−18	4

Numbering corresponds to brain regions shown in Fig. 1. Coordinates (X, Y, Z) of the clusters’ peak voxels are reported in Talairach space. Volume is mm^3^

*B* bilateral, *R* right, *L* left, *BA* Brodmann area, *OFC* orbitofrontal cortex, *vmPFC* ventromedial prefrontal cortex, *vlPFC* ventrolateral prefrontal cortex, *dmPFC* dorsomedial prefrontal cortex, *mPFC* medial prefrontal cortex

### Conjoint chronic smoking-related structural effects and acute drug-induced functional effects

**Fig. 2:**
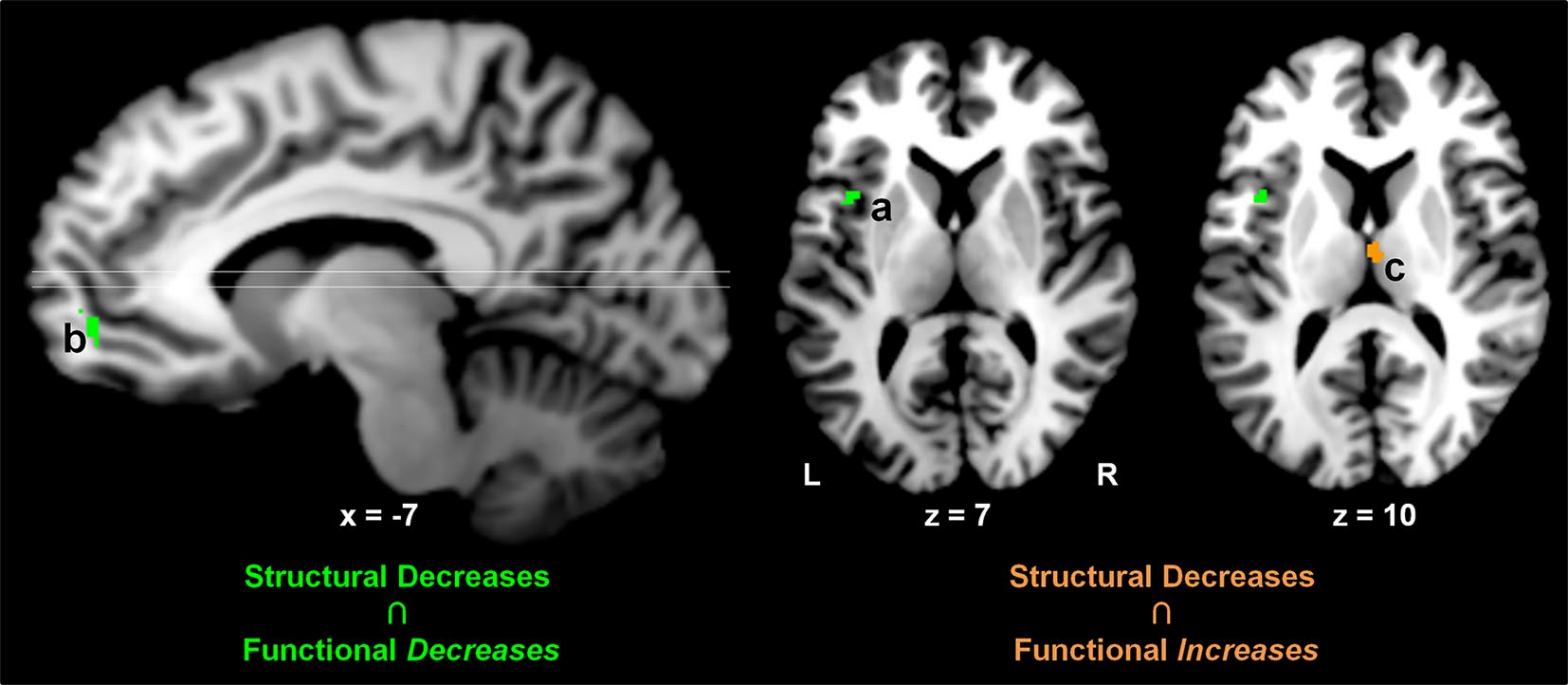
Conjoint structural and functional effects. Structural alterations (nonsmokers > smokers) overlapped with acute drug-induced activity *decreases* (baseline > drug) in the insula and ventromedial PFC (*green* **a**, **b**). Structural alterations (nonsmokers > smokers) overlapped with acute drug-induced activity *increases* (drug > baseline) in the mediodorsal thalamus (*orange* **c**). Lettering corresponds to coordinates listed in Table 2. See Additional file 1: Figures S2 and S3 for visualization of overlapping and non-overlapping regions from the structural and functional meta-analyses

To delineate regions displaying *both* structural alterations linked with chronic smoking *and* functional modulations linked with acute nicotinic agonist administration, we performed a multimodal assessment. Specifically, we conducted a conjunction analysis identifying regions showing both convergent: (1) smoking-related *structural* decreases (Fig. 1), and (2) nAChR agonist-induced *functional* decreases or increases [6]. This multimodal assessment identified overlapping structural and functional effects within the vmPFC, left insula, and MD thalamus (Fig. 2; Table 2). Specifically, whereas gray matter decreases in the vmPFC and insula overlapped with clusters of acute drug-induced functional activity *decreases* (Fig. 2, green; Additional file 1: Figure S2), gray matter decreases in MD thalamus overlapped with drug-induced activity *increases* (Fig. 2, orange; Additional file 1: Figure S3). We arrived at similar outcomes and the same conclusions when performing this multimodal assessment when considering only functional studies involving nicotine administration (i.e., excluding other nAChR agonists; Additional file 1: Figure S4) and when considering only functional results involving cigarette smokers (Additional file 1: Figure S5).

**Table 2:** Conjoint chronic smoking-related structural alterations and acute drug-induced functional activity changes: cluster coordinates

Cluster	Region		Volume	X	Y	Z
Gray matter decreases ∩ functional decreases						
a	Insula (BA 13)	L	185	−39	7	9
b	vmPFC (BA10) (superior frontal gyrus)	L	103	−9	50	−3
Gray matter decreases ∩ functional increases						
c	Thalamus (medial dorsal nucleus)	B	142	2	−14	11

Lettering corresponds to brain regions shown in Fig. 2. Coordinates (X, Y, Z) of the clusters’ peak voxels are reported in Talairach space. Volume is mm^3^

*B* bilateral, *R* right, *L* left, *BA* Brodmann area, *vmPFC* ventromedial prefrontal cortex

### Behavioral relevance of structurally impacted regions: MACM and behavioral decoding

**Fig. 3:**
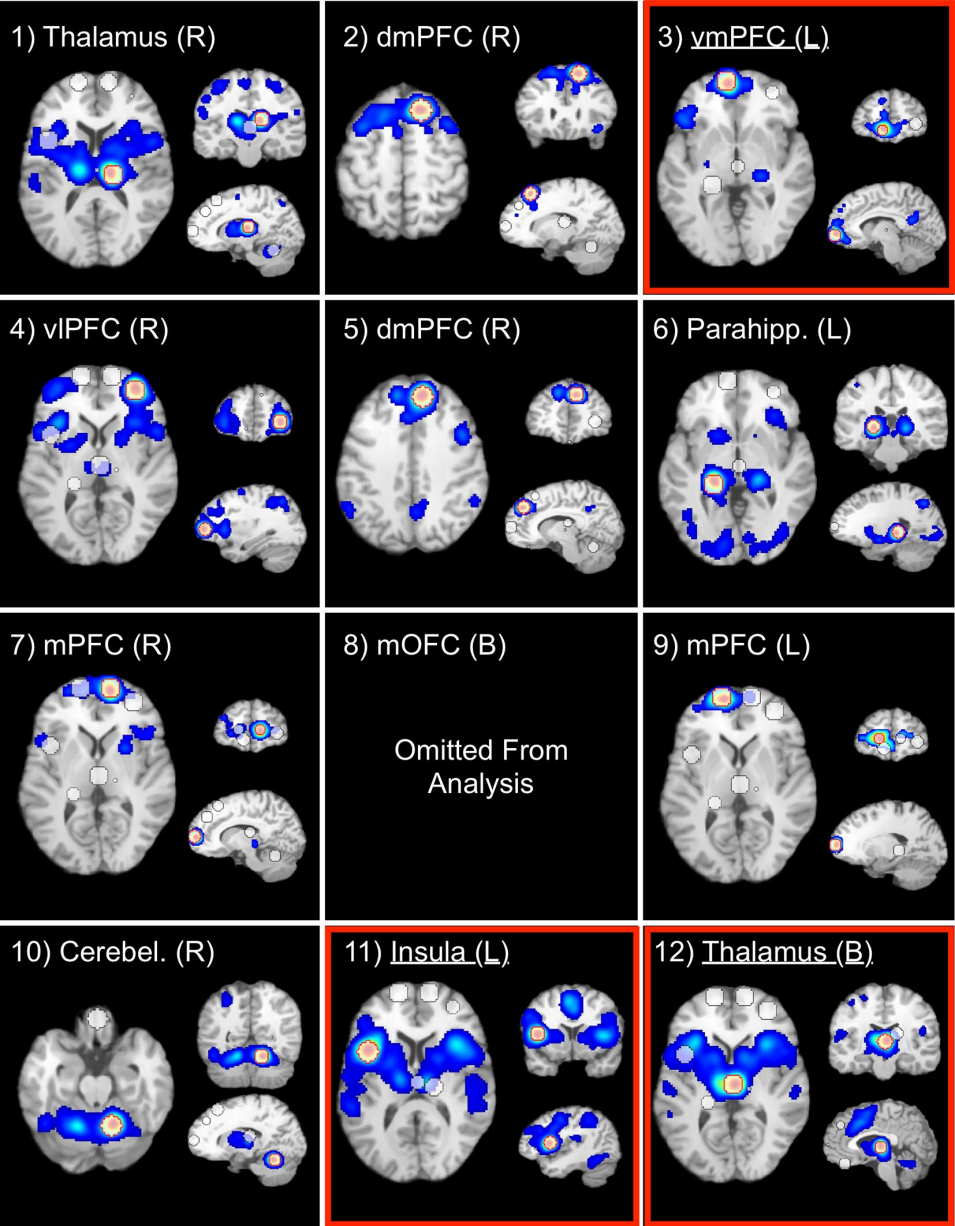
Meta-analytic connectivity modeling (MACM) maps of task-related co-activation for each structurally impacted ROI. These thresholded MACM maps (*p*_*corrected*_ < 0.05) represent voxels with an above-chance probability of co-activating with the respective seed regions (8 mm radius* spheres* centered on the voxels with maximum ALE values within each smoking-related gray matter loss region). One region (ROI 8; mOFC) failed to return a sufficient number of contrasts from the database and was omitted from further analyses. *Numbering* corresponds to that in Table 1. The seed ROI of each MACM map is outlined in *red*. MACM maps for ROIs *boxed* in *red* are discussed in the main text. See Additional file 1: Table S4 for each seed ROI’s co-activation coordinates

To enhance insight into the circuit-level consequences of smoking-related structural alterations, we first performed a MACM assessment thereby identifying clusters of convergent co-activation for each of the structurally impacted ROIs. One region (mOFC) failed to return a sufficient number of experiments from the BrainMap database and was not considered further (Additional file 1: Table S3). The remaining 11 whole-brain MACM maps represent voxels with an above-chance probability of co-activating with the seed when considering various neuroimaging tasks (Fig. 3; Additional file 1: Table S4). For example, the MACM map for: (1) the *vmPFC* seed (Fig. 3, ROI 3) indentified convergent co-activation within the posterior cingulate cortex, dmPFC, parahippocampus, and inferior frontal gyrus, (2) the *left insula* seed (Fig. 3, ROI 11) displayed notable co-activation with the right insula, posterior medial prefrontal cortex (encompassing the ACC and supplemental motor area), the thalamus, parietal cortex, and cerebellum, and (3) the *MD thalamus* seed (Fig. 3, ROI 12) encompassed the posterior medial PFC, bilateral insula, parietal cortex, and cerebellar regions. These and similarly-derived MACM maps delineating networks of task-based co-activation resemble networks identified when considering task-independent resting-state fMRI data [28, 61–64].

**Fig. 4:**
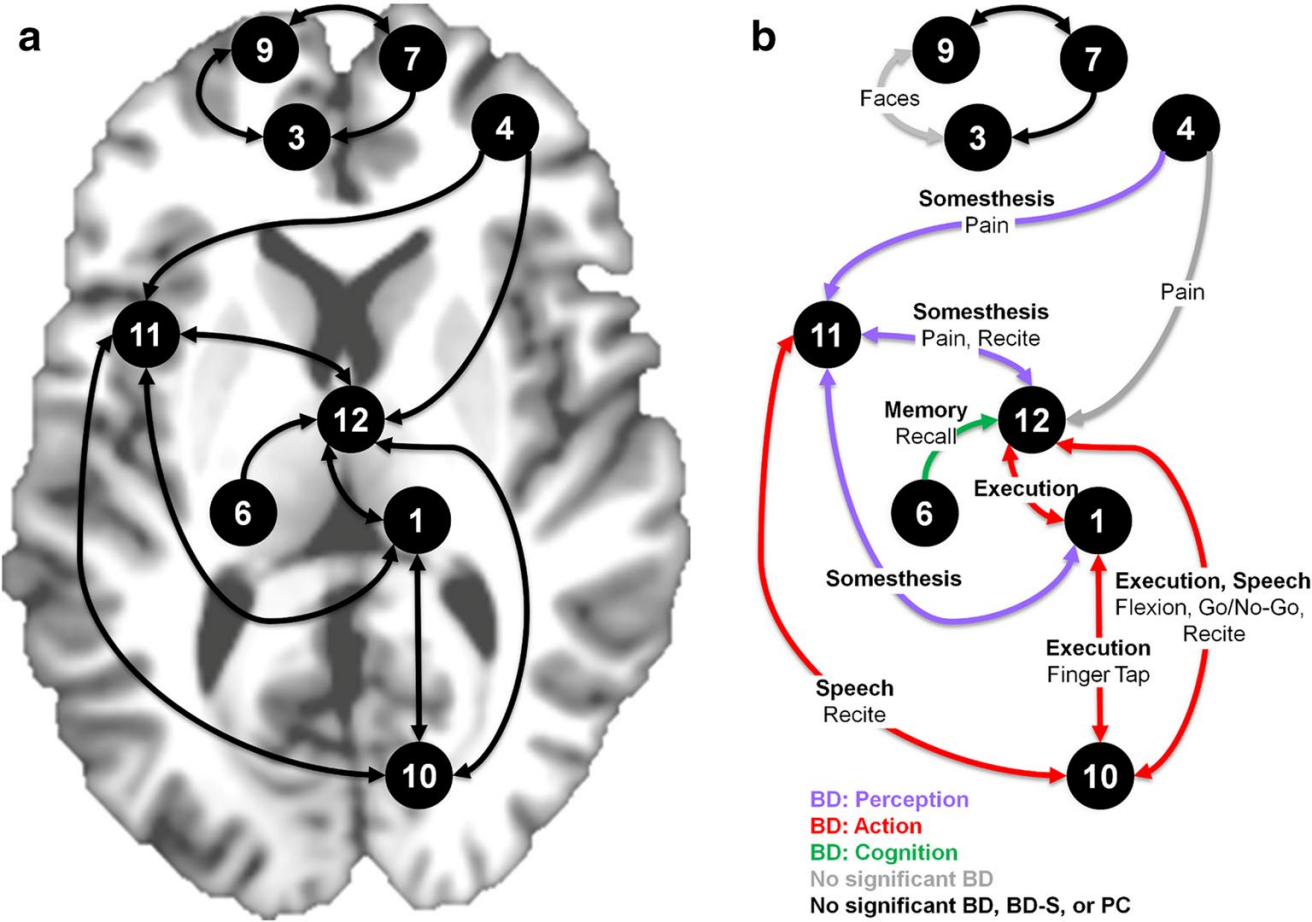
Pairs of functionally-related ROIs and associated behavioral phenomena. **a** Functionally-related ROIs are connected by paths, the directionality of which indicates that a given ROI (ending point, *arrow head*) intersected another ROIs MACM map (starting point). We note that if one ROI overlapped another ROI’s MACM map, it did not necessarily indicate that the latter ROI overlapped with the former’s MACM map. See Additional file 1: Figure S6 for an alternative representation. **b** Behavioral phenomena significantly associated with functionally-related ROIs were identified by forward and reverse inference assessments of metadata terms. In this representation, significant behavioral domains (BD) are represented by* path color* (perception [*purple*], action [*red*], cognition [*green*], no significant BD [*gray*]), significant behavioral domain subcategories (BD-S) are shown along the path in *bolded text*, and significant paradigm classes (PC) are in *standard text*. *Black paths* indicate that no significant associations were detected with any BD, BD-S, or PC

We then further assessed these co-activation maps to determine whether structurally impacted regions reflected functionally interrelated neurocircuits. Those structurally impacted ROIs that also appeared to be functionally related are summarized in Fig. 4a. In this representation, the paths between regions indicate the observation of one ROI intersecting with another ROI’s MACM map. For example, the left insula’s MACM map (Fig. 4a, ROI 11) overlapped the lateral posterior thalamus (ROI 1), cerebellum (ROI 10), and the MD thalamus (ROI 12) and vice versa (represented by double-headed arrows), whereas the left insula ROI was overlapped by the MACM map derived for the vlPFC seed (ROI 4, single-headed arrow). For the 11 ROIs, 12 unique paths were identified (out of 55; Additional file 1: Figure S6) representing pairs of regions with an above-chance probability of co-activation.

To characterize behavioral phenomena linked with these pairs of functionally-related ROIs, we performed behavioral decoding via forward and reverse inference techniques on the associated BrainMap metadata terms (BD, BD-S, and PC). Those behavioral phenomena significantly associated with co-activation of ROI pairs are represented in Fig. 4b. We highlight four observations from this assessment. First, the left insula’s co-activation with multiple other ROIs was linked with aspects of pain processing. Specifically, insula and vlPFC co-activation (Fig. 4b, path: ROI 4–11) was significantly associated with the BD *perception* (purple), the BD-S *somesthesis*-*pain*, and the PC *pain monitoring/discrimination*. Similarly, concurrent activation of the insula and MD thalamus (Fig. 4b, path: ROI 11–12) was linked with *perception*, *somesthesis*-*pain*, and *pain monitoring/discrimination* in addition to the PC *recitation/repetition (overt)*. Insula and lateral posterior thalamus co-activation (Fig. 4b, path: ROI 11–1) was associated with *perception* and *somesthesis*-*pain*. Second, the right cerebellum’s co-activation with multiple other ROIs was related to aspects of overt body movements. Specifically, cerebellum and insula co-activation (Fig. 4b, path: ROI 10–11) was associated with the BD *action* (red), the BD-S *execution*-*speech*, and the PC *recitation/repetition*. Similarly, concurrent activation of the cerebellum and MD thalamus (Fig. 4b, path: ROI 10–12) was related to *action*, *execution* (non-speech), *execution*-*speech*, and *recitation/repetition* in addition to the PCs of *flexion/extension* and *Go/No*-*Go* (i.e., inhibiting an overt body movement). Co-activation of the cerebellum and lateral posterior thalamus (Fig. 4b, path: ROI 10–1) was linked with the BD *action*, BD-S *execution* (non-speech), and PC *finger tapping/button press*. Third, co-activation of the parahippocampus and MD thalamus (Fig. 4b, path: ROI 6–11) was related to the BD *cognition* (green), the BD-S *memory*-*explicit*, and the PCs *cued*-*explicit* and *recognition/recall*. Lastly, concurrent activation of the vmPFC and mPFC (Fig. 4b, path: ROI 3–9) was associated with the PC *face monitoring/discrimination*.

## Discussion

We compiled structural MRI results to clarify the impact of cigarette smoking on gray matter integrity. Our meta-analytic results revealed convergent gray matter decreases among smokers in multiple regions including the prefrontal cortex, insula, thalamus, and cerebellum. Given that such structural measures provide limited insight into functional or behavioral implications, we subsequently performed two additional meta-analytic assessments to more fully contextualize these gray matter decreases. Indicative of a structural–functional relation, we observed via a multimodal assessment that chronic smoking-related structural effects overlapped with the acute functional effects of nAChR agonist administration in the vmPFC, left insula, and MD thalamus. Suggestive of structural-behavioral implications, we then identified pairs of structurally impacted regions that tended to co-activate across various tasks and delineated behavioral phenomena linked with such co-activation via MACM and behavioral decoding, respectively. These assessments linked the left insula’s co-activation with multiple other brain regions to pain perception, the right cerebellum’s co-activation with other regions to overt body movement, co-activation of the parahippocampus and MD thalamus with memory processes, and co-activation of medial PFC regions with face processing.

Across studies, we observed convergent smoking-related structural decreases in discrete brain regions notably in the PFC (i.e., ventromedial, ventrolateral, dorsomedial, orbitofrontal), insula, thalamus, and cerebellum. Although we can only speculate on the pathogenesis of such decreases, structural differences between smokers and nonsmokers could be products of predisposing neurobiological risk factors [65], the deleterious impact of cigarette smoking on microvascular functioning [66], neurotoxic events (e.g., oxidative stress, inflammation) associated with many of the >4000 compounds present in tobacco smoke [67], and/or be mediated directly or indirectly by nicotine itself. Focusing on the last possibility, nAChRs are ubiquitous throughout the human brain with particularly high densities in the thalamus, insula, and vmPFC [68, 69]. Widespread upregulation of nAChRs, likely related to receptor desensitization from nicotine exposure, has been observed among smokers [70, 71] and, conversely, smoking cessation associated with decreases in regional nAChR densities [72]. Evidence from preclinical models suggests neurotoxic events and protracted consequences on cholinergic neurotransmission following nicotine exposure particularly during adolescence [73–75]. As such, one plausible mechanistic account is that repeated stimulation of high densities of nAChRs by nicotine may contribute to neurotoxicity within discrete regions and/or increase the vulnerability of those regions to other volatile compounds in tobacco smoke [19] which ultimately manifest as gray matter decreases.

Speaking to such an account, we conducted a multimodal meta-analytic assessment to determine if smoking-related structural decreases overlapped with nicotinic agonist-induced functional modulations and observed conjoint effects in the vmPFC, insula, and MD thalamus. In addition to possessing relatively high nAChR densities, these regions also are critically implicated in addiction-related psychological processes. For example, the vmPFC, in concert with other regions such as the OFC and striatum, is thought to play a role in representing subjective value information, behavioral choice selection, and evaluating outcomes and alterative rewards [76–78]. The insula, a functionally heterogeneous region, is involved with monitoring the physiological state of the body, appears causally related to the initiation, maintenance, and adjustment of attentional control, and likely plays a role in maladaptive decision-making among drug addicts [79–82]. Dysfunction of the MD thalamus, a primary node within a thalamic-PFC-basal ganglia network involved in associative learning, is implicated in the transition from goal-directed action (e.g., recreational drug use) to habitual forms of responding (e.g., dysregulated drug seeking) [83, 84]. Consistent with the notion that structural alterations in these regions are a product of prolonged nicotine/tobacco exposure, group differences between smokers and nonsmokers within, for example, the insula were not detected among an adolescent/young adult participant sample with relatively lower exposure levels (0.9 ± 0.7 pack-years on average) [85] but were observed among similarly aged individuals with relatively higher exposure levels (4.0 ± 1.8 pack-years) [86]. Furthermore, multiple studies have provided evidence for dose-dependent exposure effects on gray matter integrity within the insula [18, 85] and vmPFC [19, 22, 86] as well as within the lateral PFC [14, 20] and cerebellum [23]. Widespread dose-dependent negative relations between pack-years and cortical thickness were recently corroborated in a large sample of older adults (*N* > 500) with particularly robust effects in the medial and lateral PFC [87]. Interestingly, positive relations between number of years since last cigarette and cortical thickness, suggesting potential recovery with smoking cessation, were also detected in medial PFC and posterior insula regions, amongst others [87]. Our multimodal meta-analytic outcomes add to a growing literature implicating the vmPFC, insula, and thalamus as contributors to the initiation, escalation, maintenance, and/or cessation of cigarette smoking.

Representing one objective approach for providing insight into the behavioral relevance of convergent gray matter decreases among smokers, we utilized emergent meta-analytic tools to delineate structurally impacted ROIs that tended to co-activate and their associated behavioral phenomena. Similar approaches have been used to quantitatively aid interpretation of regions identified via structural assessments or networks commonly observed via resting-state fMRI (i.e., assessments inherently lacking direct behavioral insight) [28, 33]. Although we adopted a quantitative approach to infer behavioral relevance of structural alterations, we note that our interpretations remain largely speculative until experimentally verified. We observed that co-activation of the left insula with thalamic and lateral PFC regions were generally linked with pain perception. Dysregulated pain processing is increasingly recognized as a potential barrier to smoking cessation [88, 89] and emerging evidence suggests bidirectional relations between pain and smoking behaviors [90, 91]. In laboratory assessments (e.g., cold pressor test), minimally-deprived or abstinent smokers, compared with nonsmokers, exhibit lower pain tolerance and report greater subjective experiences of pain where such pain reports positively correlate with nicotine withdrawal severity [92–94]. As such, nicotine abstinence may contribute to enhanced pain sensitivity that could maintain cigarette smoking via negative reinforcement mechanisms similar to other withdrawal symptoms [95]. Although the insula’s role in interoceptive monitoring has been well characterized [80] and higher nAChR availability in the thalamus, PFC, and cerebellum has been positively correlated with increased pain sensitivity among abstinent smokers [96], relatively little neuroimaging work has focused on the neurobiological processes that may contribute to smoking- or withdrawal-related pain dysregulation. Our meta-analytic outcomes suggest that neuroimaging investigations of pain perception among smokers, with emphasis on the putative functional interactions between the insula and MD thalamus, may be a productive avenue for future research.

We also observed that co-activation of the right cerebellum with insula and thalamic regions were generally linked with overt body movements. Acutely administered, nicotine augments performance in simple motor tasks [9] and increases cerebellar vermis activity during externally paced finger tapping [97]. On the other hand, chronic cigarette smokers perform worse than nonsmokers on neurocognitive measures of fine motor skills and postural stability [11]. Smoking-related decreases in right cerebellum gray matter, identified via an ROI-based approach [16] (i.e., a study not meeting inclusion criteria for the current meta-analysis), further corroborate our observation of convergent decreases in this region. Whereas our outcomes are consistent with the cerebellum’s critical role in motor coordination, they also align with contemporary views emphasizing the region’s contribution to cognitive processes [98, 99] and relevance to drug addiction [100, 101]. For example, we observed that co-activation of the cerebellum and MD thalamus was significantly associated with Go/No-Go paradigms that require inhibitory control. Although current neurobiological models of addiction have yet to provide an integrative account of this region’s contribution, altered cerebellar structure and function appears to be a common characteristic across drugs of abuse [100]. Our meta-analytic outcomes serve to further draw attention to the cerebellum in the context of addiction in general and cigarette smoking in particular.

Lastly, co-activation of the parahippocampus and MD thalamus was linked with memory processes and co-activation of medial PFC regions linked with face processing. Regarding the former observation, neurocognitive assessments have consistently identified visuospatial learning and memory deficits among older (>60 years) as well as middle-aged smokers (30–60 years) relative to nonsmokers [11, 12]. Regarding the latter observation, as face processing paradigms often directly or indirectly involve affective components, a plausible interpretation is that smoking-related structural effects in medial PFC regions may relate to disrupted emotional processing and/or regulation [102, 103]. Indeed, smokers tend to report elevated negative mood and a reduced ability to self-regulate such states when compared with nonsmokers [104, 105].

Our findings should be considered in light of remaining issues. First, the ALE framework is a coordinate-based meta-analytic approach that does not incorporate the size of identified clusters from the primary studies, which leads to less precise representations relative to image-based approaches [106], which, on the other hand, are themselves often less feasible. Second, we only considered gray matter decreases among smokers owing to a small number of reported experiments/foci in the literature with respect to increases. Nonetheless, neuroimaging studies have documented smoking-related volumetric increases in the caudate and putamen [17, 86], positive correlations between putamen volume and lifetime nicotine/tobacco exposure [107], and positive correlations between greater striatal volume and more intense cue-induced tobacco craving [108]. Third, we followed previous recommendations that at least 10-15 experiments should be included in an ALE meta-analysis and note that the meta-analysis identifying gray matter decreases among smokers involved 14 studies. Based on recent data simulations [43], closer to ~20 experiments has been prescribed to achieve sufficient power to detect moderately sized effects. Fourth, meta-analytic results are limited by the methodology commonly employed by the included studies. With respect to the use of T1-weighted MRIs to assess gray matter, we note recent commentaries regarding the potential for drug-induced cerebral blood flow alterations to complicate interpretation of structural outcomes [109, 110]. Given that many studies included in this meta-analysis did not report the time elapsed between last cigarette smoked and MRI data collection (Additional file 1: Table S2), this potential confounding factor cannot be ruled out. Fifth, our behavioral decoding outcomes are limited by the studies archived in the BrainMap database at the time of analysis as well as its associated taxonomy. Sixth, although some studies have begun to characterize the impact of smoking on gray matter in young adult/adolescent smokers [85, 86], the concurrent influence of marijuana [111] or alcohol use [112], and sex differences among smokers [17], as more studies accumulate regarding the influence of these and other important factors on gray matter alterations, it will become possible to better characterize the specificity of the meta-analytic effects identified herein. Lastly, it remains for future work, perhaps through multi-site longitudinal investigations, to determine whether regional structural alterations are a cause or consequence of cigarette smoking (or a combination thereof) and the extent to which such alterations recover following cessation.

In sum, cigarette smoking was associated with convergent structural decreases across studies in the PFC, insula, thalamus, and cerebellum. Some of these chronic smoking-related structural effects overlapped with regions showing acute nicotinic drug-induced functional effects. This study highlights the utility of using neuroimaging meta-analytic techniques to compile, synthesize, and inform neuroimaging investigations aiming to elucidate the neurobiological factors underlying the initiation, escalation, maintenance, and/or cessation of cigarette smoking. Collectively, our findings emphasize brain regions (e.g., vmPFC, insula, thalamus) and circuits (e.g., insula-thalamus) linked with chronic smoking, suggest neuroimaging paradigms warranting additional consideration among smokers (e.g., pain processing), and point to regions in need of further elucidation in addiction (e.g., cerebellum).

## Additional file

10.1186/s12993-016-0100-5 Supplementary tables and figures.

## Footnotes

1 Paired sample t test. One study did not report the number of females/males.

2 We note that these analyses represent an attempt to relate behavioral phenomena with identified ROIs as opposed to claiming “a unique role” of those ROIs with respect to behavior [34]. In other words, an association of “*behavioral phenomenon x to brain region y*” obtained from these analyses does not necessarily imply that activity in “*region y is limited to behavioral phenomenon x*”.
