# Supplementary material for: Chronic cigarette smoking is linked with structural alterations in brain regions showing acute nicotinic drug-induced functional modulations
Source: Behav Brain Funct. 2016 Jun 2;12:16. doi: 10.1186/s12993-016-0100-5 (PMC4890474; doi:10.1186/s12993-016-0100-5)
Supplement: Supplementary file 1 — 10.1186/s12993-016-0100-5 Supplementary tables and figures. [file 12993_2016_100_MOESM1_ESM.pdf]

SUPPLEMENTAL INFORMATION

Chronic cigarette smoking is linked with structural alterations in brain regions showing acute nicotinic agonist-induced functional modulations

Matthew T. Sutherland, Michael C. Riedel, Jessica Flannery, Julio A. Yanes, Peter T. Fox, Elliot A. Stein, and Angela R. Laird

SUPPLEMENTAL CONTENT

SUPPLEMENTAL TABLES

- **Table S1**: Participant characteristics for included studies.
- **Table S2**: Additional methodological details for included studies.
- **Table S3**: MACM seed regions: Numbers of experiments, foci, and subjects.
- **Table S4**: MACM co-activation coordinates for structurally-impacted seed regions.

SUPPLEMENTAL FIGURES

- **Figure S1**: Diagram of study selection.
- **Figure S2**: Overlapping and non-overlapping clusters from structural and functional (decreases) meta-analyses.
- **Figure S3**: Overlapping and non-overlapping clusters from structural and functional (increases) meta-analyses.
- **Figure S4**: Conjoint structural and functional effects *when considering only functional studies involving nicotine administration*.
- Figure S5: Conjoint structural and functional effects *when considering only functional studies involving cigarette smokers*.
- Figure S6: Delineating functionally interrelated ROIs.

SUPPLEMENTAL REFERENCES

<u>Table S1.</u> Participant characteristics for included studies reporting gray matter alterations among cigarette smokers versus nonsmokers.

Study	*N* Foci		Sample Size (*N* females)		Age (Range)		Smoker Characterization Details					
	Nonsmoker > Smoker	Smoker > Nonsmoker	Nonsmoker	Smoker	Nonsmoker	Smoker	Criteria	Pack Years	FTND	Years Smoking	Cigs/ Day	Time Since Last Cig
Stoeckel et al. (2015)	1	0	16 (5f)	16 (4f)	34.2 ± 7.2 (18-55)	37.9 ± 11.6 (18-55)	>10 cigs/day (6 month)	16.1 ± 12.2	4.4 ± 2.2	17.6 ± 10.5	16.8 ± 4.9	1 hr
Hanlon et al. (2014)	6	2	60 (27f)	58 (25f)	30[a] (20-49)	32[a] (20-49)	ND	12[a]	4.6[a]	NR	16[a]	1 hr
Franklin et al. (2014)	4	3	80 (39f)	80 (39f)	32[a]	33.9[a]	ND	10.5[a]	4.5[a]	14[a]	15[a]	NR
Wang et al. (2014)	3	2	20 (0f)	22 (0f)	21.8 ± 1.3 (19-28)	22.5 ± 2.5 (19-28)	>8 cigs/day (1y), ND	3.1 ± 2.6	NR	5.0 ± 2.3	11.9 ± 6.1	12 hr
Fritz et al. (2014)	16	0	659 (416f)	315 (167f)	51.5 ± 14.5	44.1 ± 11.8	self-reported regular smoker	17.8 ± 12.3	NR	26	13.2 ± 7.0	NR
Morales et al. (2012)	1	0	18 (8f)	25 (12f)	30.1 ± 2.2 (18-55)	35.4 ± 1.8 (18-55)	smoked 25 of last 30 days	11.5 ± 21.9	3.8 ± 0.4	19	14.1 ± 1.2	NR
Zhang et al. (2011)	1	1	24[b] (12f)	24[b] (12f)	36.6 ± 6.5	36.8 ± 8.0	NR	18.6 ± 7.1	5.6 ± 1.9	18.4 ± 5.8	21.4 ± 8.2	2 hr
Yu et al. (2011)	2	0	16 (0f)	16 (0f)	39.2 ± 4.5	41.6 ± 5.5	>15 cigs/day (15y), ND	21.7	7.2 ± 1.4	21.1 ± 3.9	20.6 ± 7.4	NR
Almeida et al. (2011)	3	0	36 (NR)	48 (NR)	77.3 ± 3.5	74.9 ± 4.0	>5 cigs/day (1y)	NR	NR	55.7 ± 7.4	19.0 ± 10.3	NR
Kuhn et al. (2010)[c]	1	0	21 (11f)	22 (14f)	30.9 ± 8.2	31.3 ± 7.8	NR	12.1 ± 13.2	2.8 ± 1.8	13.7 ± 8.1	13.4 ± 8.8	NR
Liao et al. (2010)	3	0	44 (10f)	44 (8f)	26.3 ± 5.8 (19-39)	28.1 ± 5.5 (19-39)	>10 cigs/day (1y), ND	NR	NR	10.4± 5.7	20.3 ± 7.7	NR
Almeida et al. (2008)	5	0	39 (14f)	39 (14f)	75.7 ± 3.2 (70-83)	75.0 ± 3.4 (70-83)	>5 cigs/day (1y)	NR	4	59[d] (40-72)	16	NR
Chen et al. (2006)	0	2	109 (109f)	11 (11f)	62.5 ± 1.4[e] (60-64)	62.5 ± 1.4[e] (60-64)	self-reported daily smoking	NR	NR	NR	NR	NR
Gallinat et al. (2006)[f]	23	0	23 (11f)	22 (10f)	30.3 ± 7.9	30.8 ± 7.5	ND	13.5 ± 13.0	2.9 ± 1.7	13.9 ± 7.3	14.5 ± 9.2	NR
Brody et al. (2004)	9	0	17 (7f)	19 (8f)	37.9 ± 12.9 (21-65)	39.5 ± 10.3 (21-65)	>20 cigs/day, ND	31.0 ± 17.9 (9-70)	5.1 ± 1.9	NR	26.2 ± 7.4 (20-40)	no smoking morning of
Overall	78	10[G]	1182	761	41.1	41.8	--	15.3	4.5	22.9	17	--

<u>Note.</u> Mean±SD reported unless otherwise indicated. *N*, number; cig, cigarette; FTND, Fagerström Test for Nicotine Dependence; y, year(s); f, females; hr, hour(s); ND, nicotine dependent; NR, not reported. [a] Smoker group characteristics calculated from smoker subgroup data reported in the original study. [b] Gray matter decreases observed in a subset of smokers (high-pack years); participant characteristics reflect this smoker subset. [c] Study assessed cortical thickness. [d] Median reported (as opposed to mean). [e] Participant characteristics are from a larger pool of participants of which only a subset were scanned. [f] Unmodulated data included in this meta-analysis (i.e., Table 3 from original study). [G] Whereas 14 studies (78 foci) reported gray matter decreases among smokers, 5 studies (10 foci) reported gray matter increases. Given the limited number of foci, gray matter increases were not considered further.

<u>Table S2.</u> Additional methodological details for included studies.

Study	Additional Details					
	Other Drug Use Screen	Scanner	Thickness, Slice (mm)	FWHM (mm)	Threshold	Software
Stoeckel et al. (2015)	interview	3T Siemens (TRIO)	1.3	8	$p < 0.05^a$	SPM8
Hanlon et al. (2014)	urine	3T Siemens (TRIO)	1	8	$p < 0.01^a$	SPM8
Franklin et al. (2014)	urine	3T Siemens (TRIO)	1	8	$p < 0.025^a$	SPM8
Wang et al. (2014)	interview	3T Siemens	1	10	$p < 0.05^a$	SPM8
Fritz et al. (2014)	interview	1.5T Siemens (Avanto)	1	12	$p < 0.05^a$	SPM8
Morales et al. (2012)	urine, interview	1.5T Siemens (Sonata)	1	8	$p < 0.05^a$	SPM8
Zhang et al. (2011)	urine, interview	3T Siemens (Allegra)	1	8	$p < 0.05^a$	FSL
Yu et al. (2011)	urine	3T Siemens (TRIO)	1	8	$p < 0.05^a$	SPM5
Almeida et al. (2011)	NR	1.5T Siemens (Symphony)	0.9	NR	$p < 0.005$ (100 voxels)	SPM5
Kuhn et al. (2010)	NR	3T Brucker (Medspec 30/100)	1.5	10	$p < 0.05^a$	Freesurfer
Liao et al. (2010)	urine, interview	3T Siemens (Allegra)	1	12	$p < 0.001$ (100 voxels)	SPM5
Almeida et al. (2008)	NR	1.5T Siemens (Symphony)	0.9	8	$p < 0.005$ (50 voxels)	SPM2
Chen et al. (2006)	NR	1.5T Philips (Gyroscan)	2	12	$p < 0.05^a$	SPM2
Gallinat et al. (2006)	interview	3T Brucker (Medspec 30/100)	1.5	12	$p < 0.05^a$	SPM2
Brody et al. (2004)	urine, interview	1.5T Siemens (Vision)	1.5	12	$p < 0.001$ (50 voxels)	SPM99

<u>Note.</u> Studies were identified via *Web of Science* (http://webofknowledge.com) and *PubMed* (http://www.pubmed.gov) database searches for peer-reviewed articles published through May 2015 with the following logical conjunction of terms: ("voxel-based morphometry" OR "morphometry" OR "gray matter density" OR "gray matter volume") AND ("nicotine" OR "tobacco" OR "cigarette" OR "smok*"). NR, not reported; T, Tesla; mm, millimeters; FWHM, full width at half maximum of spatial smoothing kernel; SPM, Statistical Parametric Mapping; FSL, FMRIB Software Library. [a] Cluster-corrected for multiple comparisons.

Table S3. Numbers of experiments, foci, and subjects utilized in the MACM assessments of each structurally-impacted ROI.

Seed ROI	Region		*N* Experiments	*N* Foci	*N* Subjects
1	thalamus (lateral posterior nucleus)	R	132	2626	1788
2	dmPFC (BA 6) (superior frontal gyrus)	R	45	709	687
3	vmPFC (BA10) (superior frontal gyrus)	L	37	445	493
4	vlPFC (BA 10) (middle frontal gyrus)	R	111	1662	1672
5	dmPFC (BA 8) (medial frontal gyrus)	R	48	611	670
6	parahippocampal gyrus	L	94	1611	1478
7	mPFC (BA10) (medial frontal gyrus)	R	43	596	584
8	medial OFC (BA 11)	B	14*	121	263
9	mPFC (BA10) (medial frontal gyrus)	L	41	736	600
10	cerebellum (dentate)	R	151	2451	1934
11	insula (BA 13)	L	229	3761	3103
12	thalamus (medial dorsal nucleus)	B	207	3564	3018

Note. We searched the BrainMap database using the *Sleuth* software application (http://www.brainmap.org/sleuth/) to identify all experiments that reported one or more activation coordinates in each of the 12 structurally-impacted seed regions. To achieve sufficient power for subsequent analyses, ROIs with less than 30 experiments were eliminated from further analysis as practiced in previously published MACM assessments (e.g., Riedel et al., 2015). Accordingly, one ROI (*; mOFC, ROI 8) was omitted from subsequent assessments. Given that this brain region is susceptible to BOLD fMRI signal loss due to magnetic field inhomogeneities (Weiskopf et al., 2007), a low number of activation coordinates archived in the database was not surprising. *N*, number. Numbering corresponds to ROIs shown in main text Figure 1 and Table 1

<u>Table S4.</u> Meta-analytic connectivity modeling (MACM) coordinates of task-related co-activation for each structurally-impacted ROI.

Seed ROI	Coactivation Cluster		Volume	x	y	z
1	Thalamus, Lateral Posterior Nucleus	R	14480	14	-20	12
	Cerebellum, Culmen	L	11600	-24	-54	-22
	Cerebellum, Culmen of Vermis	L	1024	-4	-62	-6
2	Superior Frontal (BA6)	R	24176	16	24	52
	Cingulate Gyrus (BA31)	L	4192	-8	-54	28
	Medial Frontal Gyrus (BA10)	L	1840	-6	52	12
	Superior Temporal Gyrus (BA39)	R	1608	-50	-58	26
	Superior Frontal Gyrus (BA8)	L	1472	-10	44	40
	Superior Temporal Gyrus (BA39)	R	1400	54	-56	22
	Inferior Frontal Gyrus (BA47)	R	1128	38	28	-10
	Middle Temporal Gyrus (BA21)	L	1096	-44	4	-24
	Medial Frontal Gyrus (BA10)	R	1000	10	50	6
	Precuneus (BA19)	R	960	40	-74	36
	Middle Temporal Gyrus (BA21)	R	848	54	-10	-12
3	Superior Frontal Gyrus (BA10)	L	13432	-6	54	-2
	Posterior Cingulate (BA23)	L	4472	-8	-56	18
	Inferior Frontal Gyrus (BA47)	L	4328	-44	22	-4
	Uncus, Amygdala	L	3256	-24	-8	-24
	Parahippocampal Gyrus, Amygdala	R	1560	28	-4	-18
	Lateral Geniculum Body	R	1408	22	-26	-4
	Inferior Frontal Gyrus (BA47)	R	1144	36	28	-8
	Cingulate Gyrus (BA24)	L	904	0	-6	44
	Precentral Gyrus (BA43)	R	856	58	-4	10
	Superior Frontal Gyrus (BA9)	L	744	-8	48	32
4	Middle Frontal Gyrus (BA10)	R	45072	32	48	4
	Insula (BA13)	L	22328	-34	18	6
	Medial Frontal Gyrus (BA6)	L	18192	0	16	44
	Inferior Parietal Lobule (BA40)	R	10072	42	-46	48
	Frontal Lobe, Sub-Gyral	L	9448	-34	48	2
	Superior Parietal Lobule (BA7)	L	7368	-26	-64	40
	Putamen, Lentiform Nucleus	L	3272	-24	-2	6
	Thalamus, Medial Dorsal Nucleus	R	1448	6	-22	6
	Cerebellum, Posterior Lobe, Declive	L	1256	-32	-58	-18
	Middle Frontal Gyrus (BA6)	L	936	-26	-6	54

<u>Note.</u> Table continued on next page.

<u>Table S4 (continued).</u>

Seed ROI	Coactivation Cluster		Volume	x	y	z
5	Medial Frontal Gyrus (BA8)	R	15872	10	42	36
	Middle Frontal Gyrus (BA9)	R	4000	44	8	36
	Supramarginal Gyrus (BA40)	L	3440	-52	-54	32
	Sub-lobar. Extra-Nuclear (BA47)	L	2496	-34	20	-2
	Superior Temporal Gyrus (BA13)	R	2440	40	-48	22
	Precuneus (BA7)	R	2112	6	-58	38
	Putamen, Lentiform Nucleus	L	1536	-20	4	12
	Inferior Frontal Gyrus (BA45)	R	1016	48	22	14
	Sub-lobar, Claustrum	R	872	36	6	-6
6	Parahippocampal Gyrus (BA27)	L	56072	-20	-30	-2
	Cerebellum, Posterior Lobe, Declive	R	15904	34	-54	-16
	Medial Frontal Gyrus (BA6)	L	10416	-42	4	30
	Medial Frontal Gyrus (BA6)	L	7872	-6	4	48
	Inferior Frontal Gyrus (BA9)	R	5368	44	4	32
	Precuneus (BA7)	L	4816	-20	-72	42
	Insula (BA47)	R	3832	32	24	2
	Superior Parietal Lobule (BA7)	R	2592	26	-60	46
7	Medial Frontal Gyrus (BA10)	R	13600	12	56	6
	Putamen, Lentiform Nucleus	R	5296	26	6	2
	Cerebellum, Posterior Lobe, Declive	R	1888	38	-68	-18
	Cerebellum, Anterior Lobe, Nodule	R	1840	0	-58	-28
	Inferior Frontal Gyrus (BA47)	L	1664	-46	14	0
	Cingulate Gyrus (BA32)	R	1288	2	26	36
	Occipital Lobe. Lingual Gyrus (BA18)	L	960	-2	-96	-4
	Cerebellum, Posterior Lobe, Pyramis	L	896	-12	-72	-30
	Thalamus	R	896	14	-28	-2
	Superior Temporal Gyrus (BA22)	R	888	42	-24	-8
	Cerebellum, Posterior Lobe,	L	800	-42	-56	-40
	Thalamus, Medial Dorsal Nucleus	R	792	4	-14	12
	Cerebellum, Anterior Lobe, Culmen	L	768	-46	-48	-30
	Cingulate Gyrus (BA32)	R	632	2	12	40
9	Medial Frontal Gyrus (BA10)	L	15704	-12	56	6
	Middle Temporal Gyrus (BA39)	L	3592	-46	-60	24
	Middle Frontal Gyrus (BA9)	L	2312	-46	8	38
	Cingulate Gyrus (BA32)	L	2224	-8	24	36
	Middle Temporal Gyrus (BA21)	L	1152	-56	-40	-4
	Precuneus (BA31)	L	952	-10	-58	20
	Inferior Frontal Gyrus (BA47)	L	912	-38	30	-8
	Thalamus, Medial Dorsal Nucleus	L	688	-6	-20	12

<u>Note.</u> Table continued on next page.

<u>Table S4 (continued).</u>

Seed ROI	Coactivation Cluster		Volume	x	y	z
10	Thalamus	L	123088	-14	-20	4
	Cerebellum, Anterior Lobe, Dentate	R	33808	16	-56	-20
	Medial Frontal Gyrus (BA6)	L	18520	-4	-8	56
11	Insula (BA13)	L	148280	-40	10	10
	Superior Frontal Gyrus (BA6)	L	25152	-4	6	50
	Inferior Parietal Lobule (BA40)	R	20680	38	-48	42
	Cerebellum, Anterior Lobe, Culmen	R	8176	22	-56	-22
	Cerebellum, Anterior Lobe, Culmen	L	7560	-22	-56	-24
12	Thalamus, Medial Dorsal Nucleus	R	122696	4	-18	6
	Medial Frontal Gyrus (BA6)	L	24384	-2	2	52
	Inferior Parietal Lobule (BA40)	L	8288	-34	-52	42
	Cerebellum, Posterior Lobe, Declive	R	7560	28	-56	-18
	Insula (BA13)	R	5736	46	-24	16
	Superior Parietal Lobule (BA7)	R	5712	28	-60	44
	Fusiform Gyrus, (BA37)	L	5368	-42	-56	-18

<u>Note.</u> Center of mass coordinates for co-activation clusters are reported. Given that many of the clusters' volumes were relatively large, the table does not adequately capture all regions of co-activation (for visualization of co-activation results, see main text Figure 3). One region (mOFC, ROI 8) failed to return a sufficient number of experiments from the database and was omitted from the table. Numbering corresponds to ROIs shown in main text Figure 1 and Table 1. Coordinates (x, y, z) are reported in Talairach space. Volume is mm^3. B, bilateral; R, right; L, left; BA, Brodmann area.

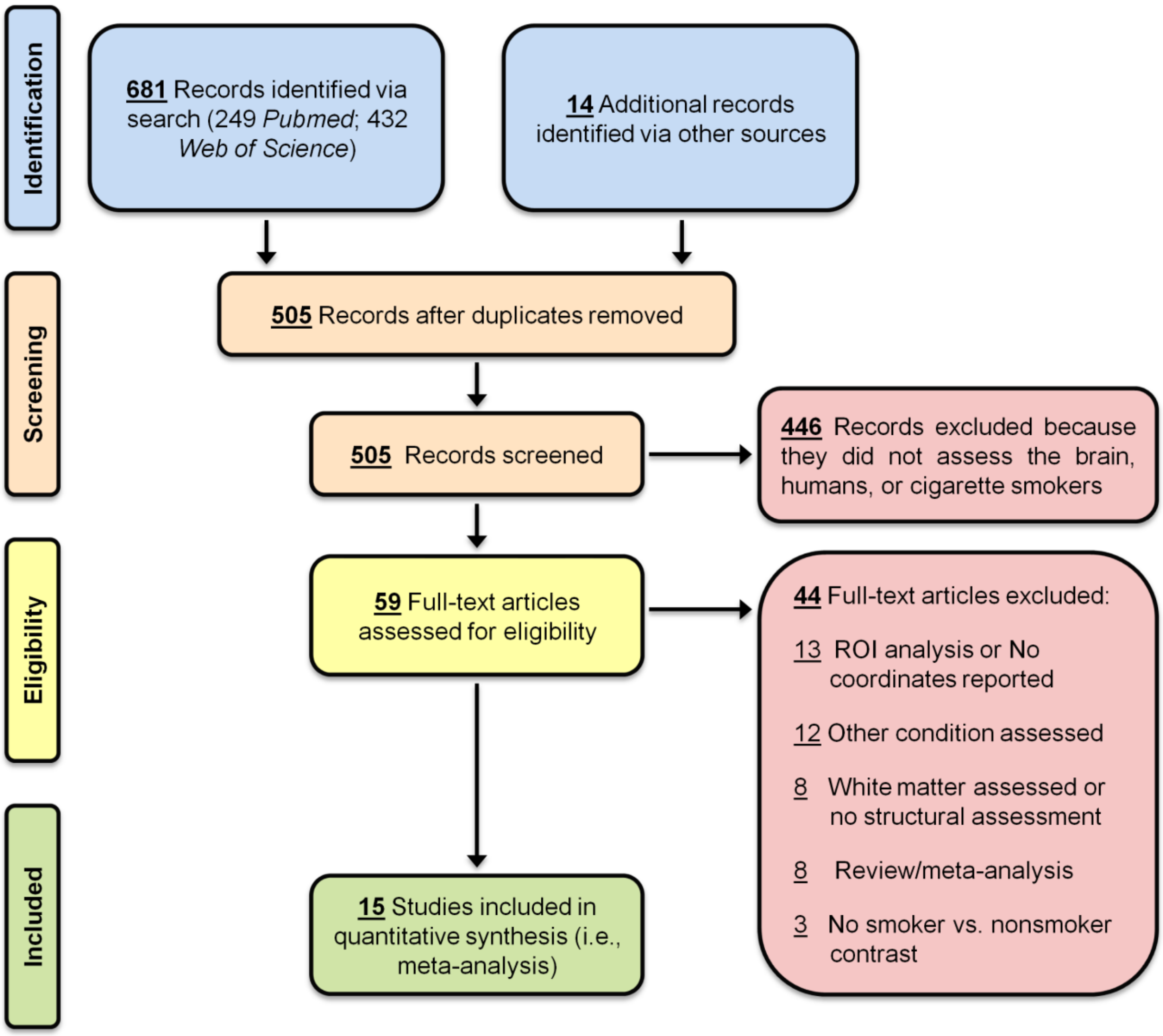

Figure S1. Diagram of study selection process (structural meta-analysis). We first searched the *PubMed* (http://www.pubmed.gov) and *Web of Science* (http://webofknowledge.com) databases for peer-reviewed articles with the following logical conjunction of terms: (“voxel-based morphometry” OR “morphometry” OR “gray matter density” OR “gray matter volume”) AND ("nicotine" OR “tobacco” OR "cigarette" OR "smok*"). We identified additional candidate publications through *other sources* by consulting the bibliographies of recent review articles, and also tracking the references of and citations to relevant papers. This literature search was conducted on papers published (or e-ahead published) through August 2015. After removing duplicates, records were screened to remove those that were not relevant to the current research question (i.e., we removed studies that did not investigate the brain, used animal models, or did not consider cigarette smokers). Of the 59 full-text articles reviewed for eligibility, 44 were excluded because the study: involved an ROI analysis or did not report coordinates of grey matter alterations, assessed other conditions in addition to cigarette smoking (e.g., schizophrenia, mental illness, poly-substance abuse, alcohol dependence), assessed white matter or brain function, was a review article or meta-analysis, or did not involve a smoker versus non-smoker contrast (e.g., within-subjects design among smokers, nicotine prenatal exposure).

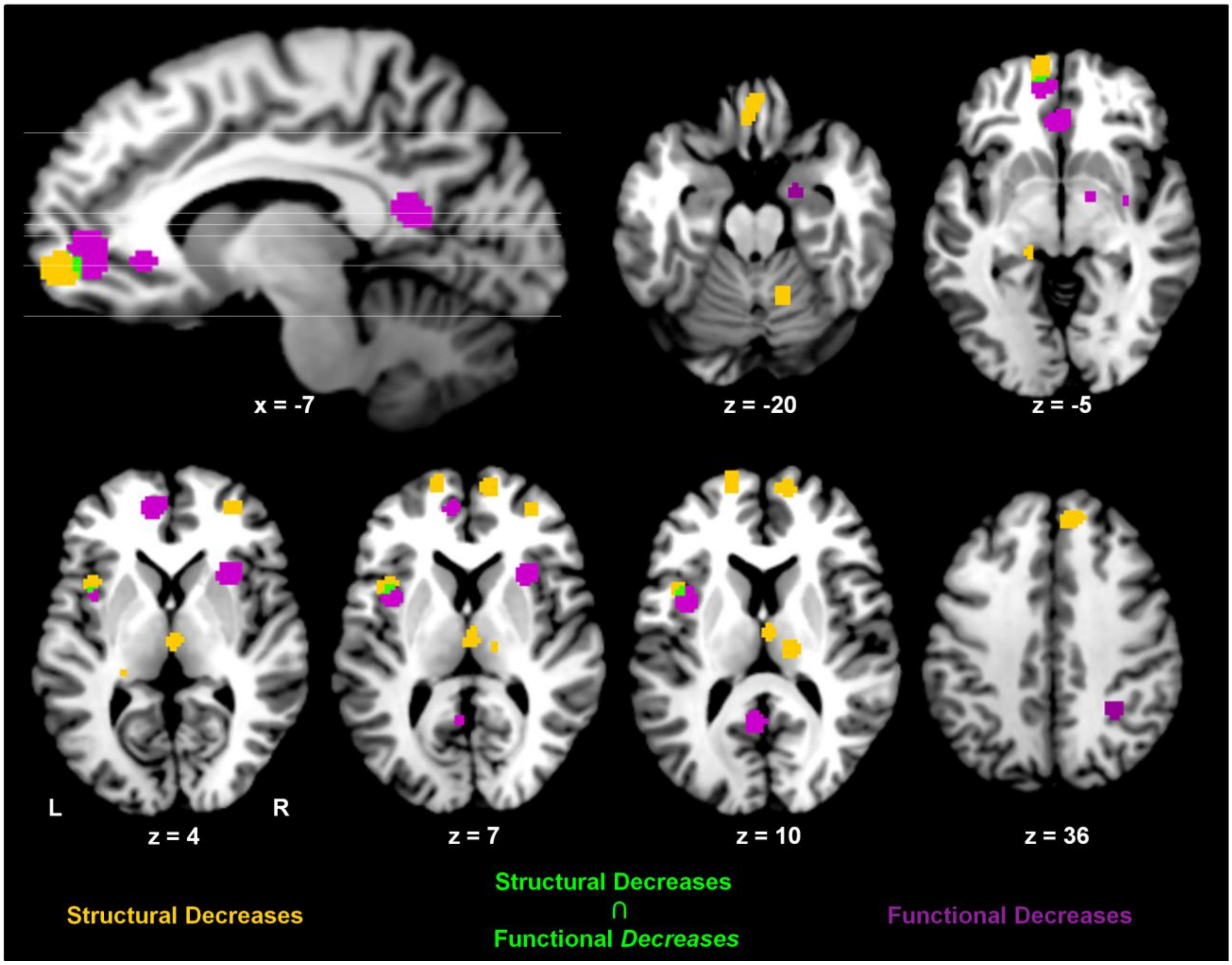

<u>Figure S2.</u> Multimodal meta-analytic assessment: Visualization of overlapping and *non-overlapping* regions associated with smoking-related structural alterations and acute drug-induced functional activity *decreases*. Gray matter decreases (**yellow**: nonsmokers > smokers; also shown in main text Figure 1 and Table 1) overlapped (**green**; also shown in main text Figure 2 and Table 2) with drug-induced functional activity decreases (**purple**; baseline > drug; reported in Sutherland et al., 2015) in the left insula and vmPFC. See Sutherland et al., (2015) for full characterization of convergent activity decreases associated with acute nAChR agonist administration.

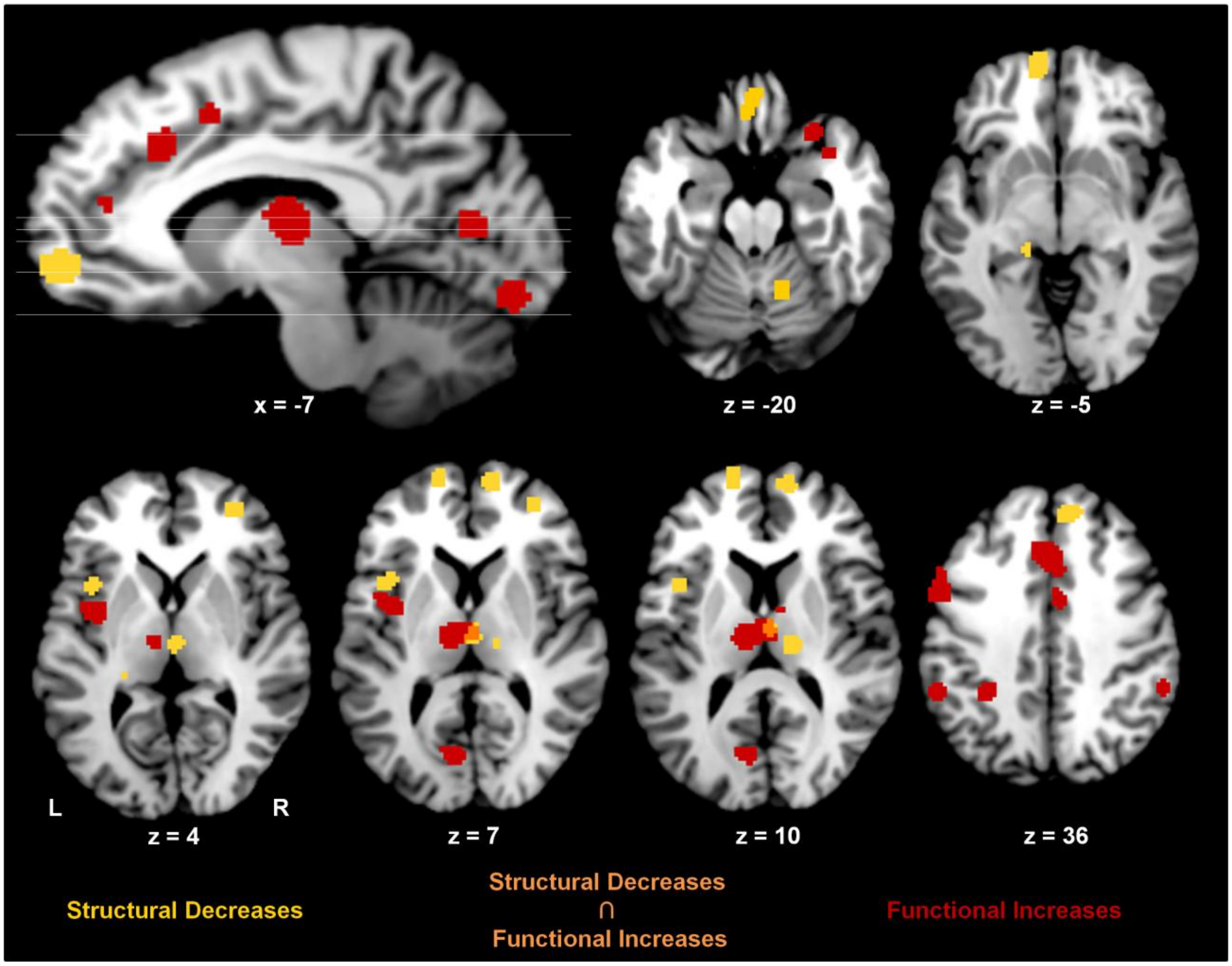

Figure S3. **Multimodal meta-analytic assessment: Visualization of overlapping and *non-overlapping* regions associated with smoking-related structural alterations and acute drug-induced functional activity *increases*.** Gray matter decreases (**yellow**: nonsmokers > smokers; also shown in main text Figure 1 and Table 1) overlapped (**orange**; also shown in main text Figure 2 and Table 2) with drug-induced functional activity increases (**red**; drug > baseline; reported in Sutherland et al., [2015]) in the MD thalamus. See Sutherland et al., (2015) for full characterization of convergent activity increases associated with acute nAChR agonist administration.

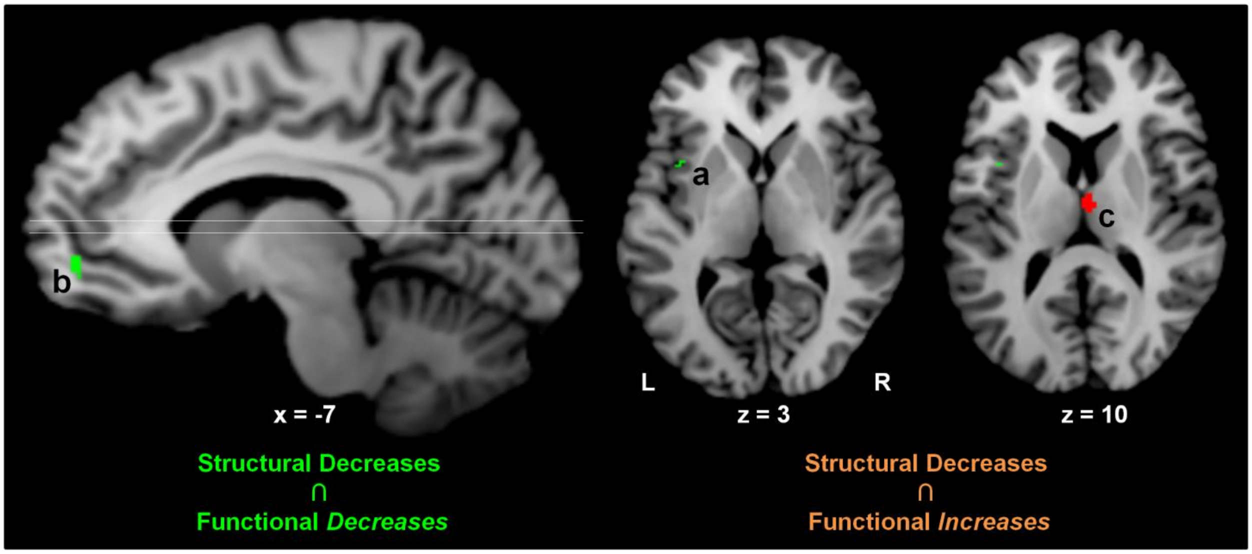

<u>Figure S4</u>. Conjoint structural and functional effects <u>*when considering only functional studies involving nicotine administration*</u>. We note that the previously published functional meta-analyses leveraged to identify structural/functional overlap in Figure 2 of the main text included studies involving multiple nAChR agonists (i.e., nicotine, varenicline and 3-(2,4-dimethoxtbensylidene)-anabaseine). For those functional meta-analyses, we identified 38 pharmacologic fMRI studies that assessed the acute effects of nAChR agonist administration (364 foci, from 77 contrasts; activity decreases: 28 studies, 179 foci, 39 contrasts; activity increases: 26 studies, 185 foci, 38 contrasts) and coded those studies according to nAChR manipulation method as involving either direct pharmacologic administration (26 studies: 263 foci, 51 contrasts) or cigarette smoking (12 studies: 101 foci, 28 contrasts). Pharmacologic administration methods employed were *nicotine delivery strategies* (transdermal patch [n=7 studies], nasal spray [n=7], buccal gum [n=5], subcutaneous injection [n=2], and buccal lozenge [n=1]), *oral varenicline* (n=3; an alpha4beta2 nAChR partial agonist/alpha7 full agonist), *or oral 3-(2,4-dimethoxtbensylidene)-anabaseine* (n=1; an alpha7 nAChR partial agonist). Thus, of the 38 pharmacological fMRI studies included in those functional meta-analyses, 4 involved nAChR agonists other than nicotine (functional activity increases: 21 foci, 5 contrasts; function activity decreases: 17 foci, 5 contrasts). The rationale for including a range of nicotine delivery methods as well as multiple nAChR agonist (which, although different drugs, are all of the same drug class as they have similar chemical structures and mechanisms of action/biological targets) in the meta-analysis, is to increase generalizability.

To determine if the inclusion of drugs other than nicotine exerted an undue influence on our previously reported outcomes, we performed an ancillary multimodal assessment involving only studies examining nicotine. The ancillary functional meta-analyses involving only studies characterizing nicotine administration, consisted of 24 studies (158 foci, 34 contrasts) reporting activity decreases and 22 studies (168 foci, 33 contrasts) reporting activity increases. Structural alterations (nonsmokers > smokers) overlapped with acute nicotine-induced activity decreases (baseline > drug) in the left insula (**green, a:** volume = 38mm^3, X = -39, Y = 5, Z = 5) and vmPFC (**green, b:** volume = 103mm^3, X = -13, Y = 51, Z = -1). Structural alterations (nonsmokers > smokers) overlapped with acute drug-induced activity increases (drug > baseline) in the mediodorsal thalamus (**orange, c**: volume = 291mm^3, X = 3, Y = -9, Z = 13). These outcomes suggest that the inclusion of studies characterizing nAChR agonists other than nicotine did not exert an undue influence on the results presented in Figure 2 of the main text and our interpretations thereof.

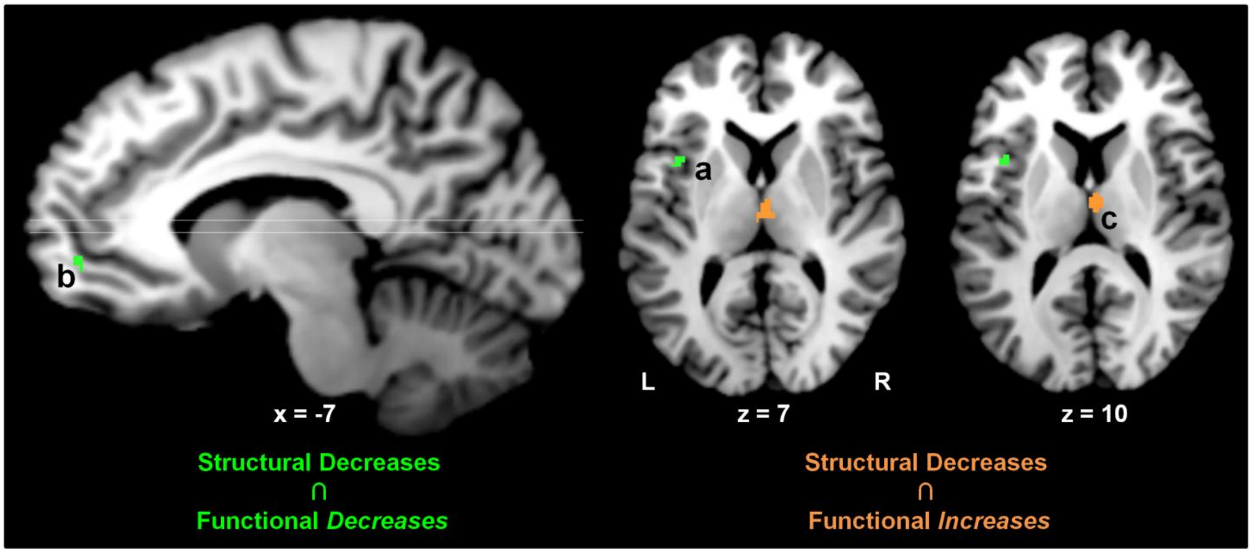

Figure S5. Conjoint structural and functional effects *when considering only functional studies involving cigarette smokers*. We note that the previously published functional meta-analyses leveraged to identify structural/functional overlap in Figure 2 of the main text included studies involving both smokers and nonsmokers. For those functional meta-analyses, we identified 38 pharmacologic fMRI studies that assessed the acute effects of nAChR agonist administration (364 foci, from 77 contrasts; activity decreases: 28 studies, 179 foci, 39 contrasts; activity increases: 26 studies, 185 foci, 38 contrasts) in studies involving both smokers (27 studies, 260 foci, 54 contrasts) and nonsmokers (11 studies, 102 foci, 22 contrasts).

To determine if the inclusion of studies involving nonsmokers exerted an undue influence on the outcomes reported in Figure 2 of the main text, we performed an ancillary multimodal assessment involving only functional studies of cigarette smokers. The ancillary functional meta-analyses involving only studies characterizing drug effects among smokers consisted of 20 studies (135 foci, 28 contrasts) reporting activity decreases and 19 studies (125 foci, 26 contrasts) reporting activity increases. Structural alterations (nonsmokers > smokers) overlapped with acute drug-induced activity decreases (baseline > drug) in the left insula (**green, a:** volume = 185mm^3, X = -39, Y = 9, Z = 13) and vmPFC (**green, b:** volume = 45mm^3, X = -11, Y = 51, Z = -1). Structural alterations (nonsmokers > smokers) overlapped with acute drug-induced activity increases (drug > baseline) in the mediodorsal thalamus (**orange, c**: volume = 337mm^3, X = 3, Y = -9, Z = 13). These outcomes suggest that the inclusion of studies involving nonsmokers did not exert an undue influence on the results presented in Figure 2 of the main text and our interpretations thereof.

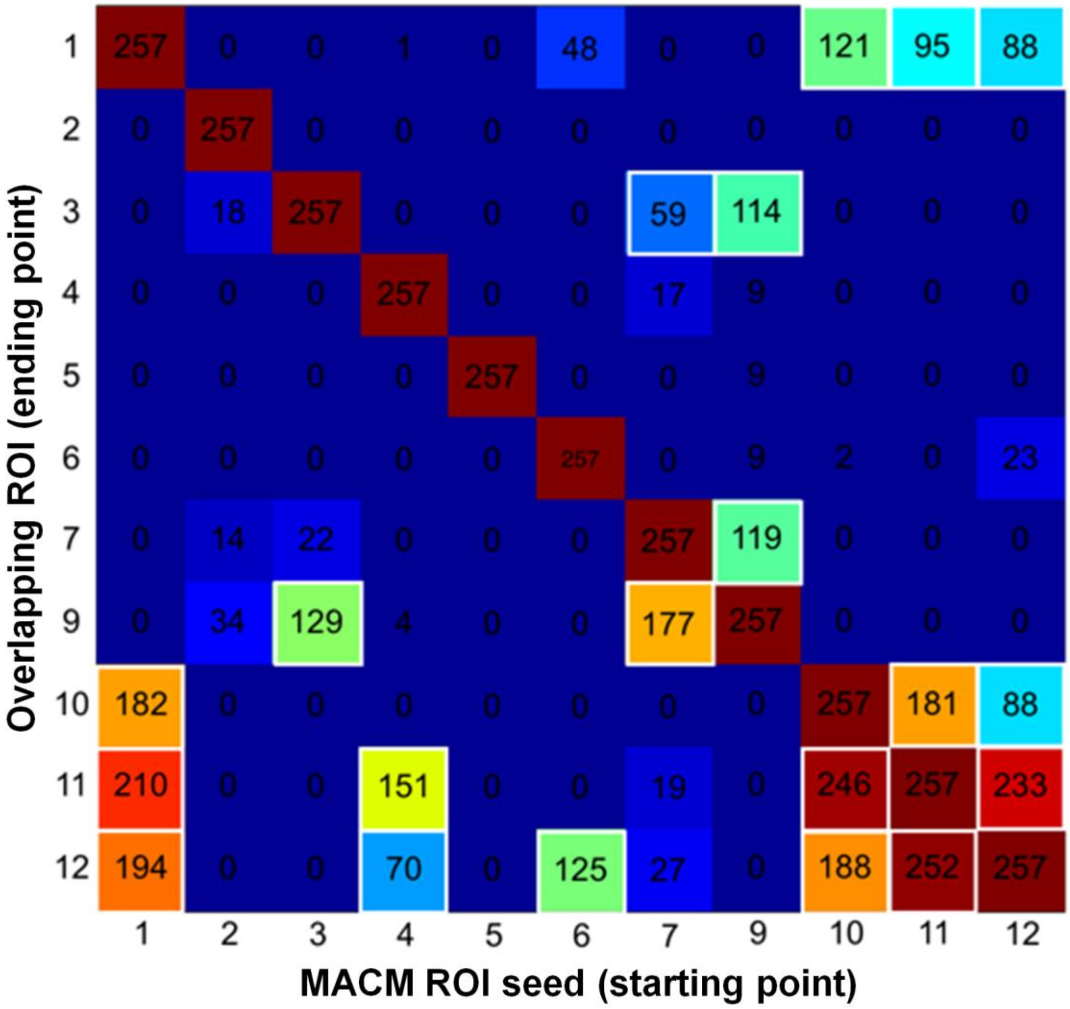

<u>Figure S6.</u> Delineating functionally related ROIs. We quantified the degree to which one ROIs MACM map (starting point) intersected with any of the other structurally-impacted ROIs (ending point). If one ROI's MACM map overlapped at least 50 voxels of any other ROI, those two regions were considered to be functionally related. The plot above depicts the numbers of overlapping voxels when considering each pair of ROIs. The *x-axis* designates the structurally-impacted ROI that was used as the seed region in a given MACM assessment and the *y-axis* represents the ROIs with which that map could overlap. Those ROIs considered functionally interrelated are boxed in white. For example, the MACM map for ROI 1 overlapped ROIs 10, 11, and 12. In total, there were 55 unique paths (one side of the diagonal). We note that if one ROI overlapped another ROI's MACM map, it did not necessarily indicate that the latter ROI would overlap the former's MACM map (i.e., a path from ROI 4 to ROI 12 does not indicate a path from ROI 12 to ROI 4). The values on the diagonal represent overlap with the seed itself. See main text Figure 4 for an alternative visualization.

SUPPLEMENTAL REFERENCES

Almeida OP, Garrido GJ, Alfonso H, Hulse G, Lautenschlager NT, Hankey GJ, Flicker L (2011) 24-month effect of smoking cessation on cognitive function and brain structure in later life. Neuroimage 55:1480-1489.

Almeida OP, Garrido GJ, Lautenschlager NT, Hulse GK, Jamrozik K, Flicker L (2008) Smoking is associated with reduced cortical regional gray matter density in brain regions associated with incipient Alzheimer disease. The American journal of geriatric psychiatry : official journal of the American Association for Geriatric Psychiatry 16:92-98.

Brody AL, Mandelkern MA, Jarvik ME, Lee GS, Smith EC, Huang JC, Bota RG, Bartzokis G, London ED (2004) Differences between smokers and nonsmokers in regional gray matter volumes and densities. Biol Psychiatry 55:77-84.

Chen X, Wen W, Anstey KJ, Sachdev PS (2006) Effects of cerebrovascular risk factors on gray matter volume in adults aged 60-64 years: a voxel-based morphometric study. Psychiatry Res 147:105-114.

Franklin TR, Wetherill RR, Jagannathan K, Johnson B, Mumma J, Hager N, Rao H, Childress AR (2014) The effects of chronic cigarette smoking on gray matter volume: influence of sex. PLoS One 9:e104102.

Fritz HC, Wittfeld K, Schmidt CO, Domin M, Grabe HJ, Hegenscheid K, Hosten N, Lotze M (2014) Current smoking and reduced gray matter volume-a voxel-based morphometry study. Neuropsychopharmacology 39:2594-2600.

Gallinat J, Meisenzahl E, Jacobsen LK, Kalus P, Bierbrauer J, Kienast T, Witthaus H, Leopold K, Seifert F, Schubert F, Staedtgen M (2006) Smoking and structural brain deficits: a volumetric MR investigation. The European journal of neuroscience 24:1744-1750.

Hanlon CA, Owens MM, Joseph JE, Zhu X, George MS, Brady KT, Hartwell KJ (2014) Lower subcortical gray matter volume in both younger smokers and established smokers relative to non-smokers. Addict Biol.

Kuhn S, Schubert F, Gallinat J (2010) Reduced Thickness of Medial Orbitofrontal Cortex in Smokers. Biological Psychiatry 68:1061-1065.

Liao Y, Tang J, Liu T, Chen X, Hao W (2012) Differences between smokers and non-smokers in regional gray matter volumes: a voxel-based morphometry study. Addict Biol 17:977-980.

Morales AM, Lee B, Hellemann G, O'Neill J, London ED (2012) Gray-matter volume in methamphetamine dependence: cigarette smoking and changes with abstinence from methamphetamine. Drug Alcohol Depend 125:230-238.

Riedel MC, Ray KL, Dick AS, Sutherland MT, Hernandez Z, Fox PM, Eickhoff SB, Fox PT, Laird AR (2015) Meta-analytic connectivity and behavioral parcellation of the human cerebellum. Neuroimage 117:327-342.

Stoeckel LE, Chai XJ, Zhang J, Whitfield-Gabrieli S, Evins AE (2015) Lower gray matter density and functional connectivity in the anterior insula in smokers compared with never smokers. Addict Biol.

Sutherland MT, Ray KL, Riedel MC, Yanes JA, Stein EA, Laird AR (2015) Neurobiological impact of nicotinic acetylcholine receptor agonists: an activation likelihood estimation meta-analysis of pharmacologic neuroimaging studies. Biol Psychiatry 78:711-720.

Wang K, Yang J, Zhang S, Wei D, Hao X, Tu S, Qiu J (2014) The neural mechanisms underlying the acute effect of cigarette smoking on chronic smokers. PLoS One 9:e102828.

Weiskopf N, Hutton C, Josephs O, Turner R, Deichmann R (2007) Optimized EPI for fMRI studies of the orbitofrontal cortex: compensation of susceptibility-induced gradients in the readout direction. Magma 20:39-49.

Yu R, Zhao L, Lu L (2011) Regional gray and white matter changes in heavy male smokers. PLoS One 6:e27440.

Zhang X, Salmeron BJ, Ross TJ, Geng X, Yang Y, Stein EA (2011) Factors underlying prefrontal and insula structural alterations in smokers. Neuroimage 54:42-48.
